# A systematic review and meta-analysis evaluating the impact of antibiotic use on the clinical outcomes of cancer patients treated with immune checkpoint inhibitors

**DOI:** 10.3389/fonc.2023.1075593

**Published:** 2023-03-02

**Authors:** Athéna Crespin, Clément Le Bescop, Jean de Gunzburg, Fabien Vitry, Gérard Zalcman, Julie Cervesi, Pierre-Alain Bandinelli

**Affiliations:** ^1^ Da Volterra, Paris, France; ^2^ Department of Thoracic Oncology and CIC1425, Institut du Cancer AP-HP, Nord, Hôpital Bichat-Claude Bernard, AP-HP, Université de Paris, Paris, France; ^3^ U830 Institut National de la Santé et de la Recherche Médicale (INSERM) “Cancer, Heterogeneity, Instability and Plasticity” Curie Institute, Paris, France

**Keywords:** systematic review, meta-analysis, cancer, antibiotics, microbiome, microbiota, immune checkpoint inhibitors, immunotherapies

## Abstract

**Background:**

Immune checkpoint inhibitors (ICIs) have considerably improved patient outcomes in various cancer types, but their efficacy remains poorly predictable among patients. The intestinal microbiome, whose balance and composition can be significantly altered by antibiotic use, has recently emerged as a factor that may modulate ICI efficacy. The objective of this systematic review and meta-analysis is to investigate the impact of antibiotics on the clinical outcomes of cancer patients treated with ICIs.

**Methods:**

PubMed and major oncology conference proceedings were systematically searched to identify all studies reporting associations between antibiotic use and at least one of the following endpoints: Overall Survival (OS), Progression-Free Survival (PFS), Objective Response Rate (ORR) and Progressive Disease (PD) Rate. Pooled Hazard Ratios (HRs) for OS and PFS, and pooled Odds Ratios (ORs) for ORR and PD were calculated. Subgroup analyses on survival outcomes were also performed to investigate the potential differential effect of antibiotics according to cancer types and antibiotic exposure time windows.

**Results:**

107 articles reporting data for 123 independent cohorts were included, representing a total of 41,663 patients among whom 11,785 (28%) received antibiotics around ICI initiation. The pooled HRs for OS and PFS were respectively of 1.61 [95% Confidence Interval (CI) 1.48-1.76] and 1.45 [95% CI 1.32-1.60], confirming that antibiotic use was significantly associated with shorter survival. This negative association was observed consistently across all cancer types for OS and depending on the cancer type for PFS. The loss of survival was particularly strong when antibiotics were received shortly before or after ICI initiation. The pooled ORs for ORR and PD were respectively of 0.59 [95% CI 0.47-0.76] and 1.86 [95% CI 1.41-2.46], suggesting that antibiotic use was significantly associated with worse treatment-related outcomes.

**Conclusion:**

As it is not ethically feasible to conduct interventional, randomized, controlled trials in which antibiotics would be administered to cancer patients treated with ICIs to demonstrate their deleterious impact *versus* control, prospective observational studies and interventional trials involving microbiome modifiers are crucially needed to uncover the role of microbiome and improve patient outcomes. Such studies will reduce the existing publication bias by allowing analyses on more homogeneous populations, especially in terms of treatments received, which is not possible at this stage given the current state of the field. In the meantime, antibiotic prescription should be cautiously considered in cancer patients receiving ICIs.

**Systematic review registration:**

https://www.crd.york.ac.uk/prospero/, identifier CRD42019145675.

## 1 Introduction

Cancer immunotherapy targeting immune checkpoints has revolutionized cancer management and resulted in significant improvement in patient outcomes in a large array of cancers (1). Currently approved immune checkpoint inhibitors (ICIs) include monoclonal antibodies targeting programmed cell death protein 1 (anti-PD-1) and its ligand (anti-PD-L1), as well as cytotoxic T-lymphocyte-associated protein 4 (anti-CTLA-4). Furthermore, numerous molecules targeting other immune checkpoints are currently being evaluated in clinical trials and could soon enrich the list of authorized ICIs. Besides, the indications of approved products are increasingly expanded to new cancer types and earlier lines of treatment^1^.

This significant and steadily increasing use of ICIs and the variation of response between patients warrant attention to the factors that mitigate their efficacy. Only between 15 and 60% of patients, depending on cancer types, do respond to ICI treatment (1, 2), which leaves a wide range of patients who do not fully benefit from ICIs. In non-small cell lung cancer (NSCLC), one of the first cancers for which ICIs were authorized, only 15 to 30% of patients seem to achieve a durable benefit from ICIs (1, 3, 4).

In recent years, the gut microbiome has been increasingly discussed as playing a crucial role in the education and development of major components of the host’s immune system, and therefore in a certain number of health conditions and diseases (5). The role of the gut microbiome in modulating or predicting the effectiveness of ICIs has also been highlighted in recent papers (6–8). Several studies have identified gut bacteria that could be associated with good or poor clinical response in the fecal microbiome of cancer patients treated with ICIs. They have even shown that fecal microbiota transplantation (FMT) from patients responding to ICIs into germ-free or antibiotic-treated mice modulated the response of mice tumors to ICI treatment (6–8).

Cancer patients are particularly vulnerable to bacterial infections and antibiotics (ABX) are often used in the clinical practice. ABX are known to induce profound changes to the gut microbiome and to disrupt the balance between the various bacterial groups, genera and species normally found in each healthy individual. Microbiome disruption, called dysbiosis, can last for several weeks or even months after ABX intake (9, 10), and alter key functions of the microbiome (11). The relationship between ABX use and ICI efficacy is therefore increasingly studied in clinical practice. ABX exposure was notably shown in numerous retrospective and prospective studies to adversely influence the clinical outcomes of patients suffering from different types of cancer treated with ICIs (12–14). Sixteen meta-analyses were published on the subject and consistently concluded on a damaging impact of ABX use on the clinical outcomes of cancer patients treated with ICIs (15–30), yet only 48 cohorts (12,794 patients) were included in the most comprehensive meta-analysis (23), leaving a large part of the literature unexploited.

By including in the present meta-analysis a total of 107 articles reporting clinical data based on ABX exposure on 123 independent cohorts, for a total of 41,663 patients, we aimed to exhaustively cover the literature of the field and to provide novel analyses that were not performed in previously published meta-analyses. In particular, the impact of ABX use on treatment-related outcomes such as Objective Response Rate (ORR) and Progressive Disease (PD) rate has been poorly investigated to date, with few articles included in the meta-analyses having performed such analyses. Also, the potential differential effect of ABX use depending on the cancer type has not been investigated in as many cancer types as possible, and, for instance, the impact of ABX use in urothelial carcinoma (UC), in which ICIs are increasingly used, has never been conclusively examined. Hopefully, our findings will help improve the understanding of the links between ABX use and ICI efficacy, optimize individualized clinical care during cancer immunotherapy and benefit patient prognosis.

This meta-analysis aims to answer the following questions: is the use of ABX before and/or during an anti-PD-L(1)-based treatment associated with a modification of the response to treatment and survival in cancer patients? Are there elements related to the cancer, the ABX therapy itself and/or the time window of ABX exposure relative to ICI initiation that could modulate this impact and help physicians issue best practice recommendations?

## 2 Materials and methods

### 2.1 Registration

The meta-analysis protocol was submitted to the International Prospective Register of Systematic Reviews CRD42019145675URL : https://www.crd.york.ac.uk/prospero/ and the research work was conducted according to the Preferred Reporting Items for Systematic Reviews and Meta-Analyses (PRISMA) guidelines (31).

### 2.2 Data sources and literature search strategy

**Figure 1:**
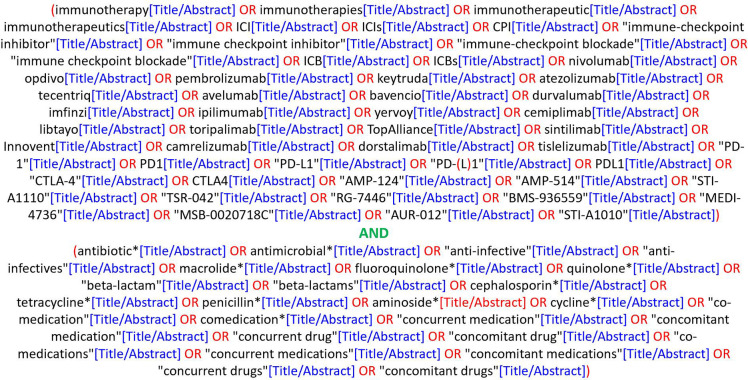
Literature query used on PubMed.

A systematic literature search was performed using MEDLINE (through PubMed) and a comprehensive query (**Figure 1**) in order to retrieve all relevant studies published until September 15, 2022 and reporting data on the associations between ABX use and the clinical outcomes of cancer in patients treated with anti-PD-(L)1-based treatments. In order to include the largest possible patient population, no filters for language (although the query was submitted in English) or year of publication were applied. Besides, proceedings of major oncology conferences held between 2017 and 2022 were also screened to identify unpublished studies that could be included, thus minimizing publication bias, using the following keywords: antibiotic, antibiotics, antimicrobial, antimicrobials, anti-infective, anti-infectives. Such conferences were the European Lung Cancer Congress (ELCC) and the World Conference on Lung Cancer (WCLC), as well as annual meetings from the American Association for Cancer Research (AACR), the American Society of Clinical Oncology (ASCO), the European Society for Medical Oncology (ESMO), the International Association for the Study of Lung Cancer (IASLC) and the Society for Immunotherapy of Cancer (SITC). Any relevant article references were also screened for additional studies.

### 2.3 Study selection

Studies were included in the present meta-analysis if they fulfilled the following criteria: 1) study subjects were patients diagnosed with any type of cancer and treated with anti-PD-(L)1 agents, either as monotherapy or in combination with other anticancer treatments, 2) ABX-exposed patients received ABX before and/or after the initiation of and/or during immunotherapy, regardless of ABX class, route of administration and duration of use, 3) ABX-unexposed patients (the control group) did not receive ABX within the defined timeframes, and 4) studies provided data, suitably formatted for inclusion, on the associations between ABX use and at least one outcome retained for this meta-analysis, namely Overall Survival (OS), Progression-Free Survival (PFS), ORR and PD.

If several studies were redundant (*i.e.* they reported data on overlapping patient populations, which was identified by looking at patient recruitment centers and study periods), the most recent study was selected for inclusion in the meta-analysis.

The literature screening was independently conducted by two reviewers who consulted with a third author to resolve any discrepancy.

### 2.4 Data extraction

Using a standardized data extraction spreadsheet, the following data were collected from each of the included study, when available: first author’s name, publication year, publication type (full-text article, poster or abstract), country, patient and cancer characteristics (*i.e*. number of patients included, histology, cancer stage, Eastern Cooperative Oncology Group performance score (ECOG PS)), immunotherapy characteristics (ICI type, treatment scheme and line of treatment), ABX treatment characteristics (number of ABX users, ABX exposure time window (TW), indication, class, route of administration and duration of use) and outcomes of interest based on ABX exposure. Authors were contacted when crucial data, such as the number of ABX users, were missing in a study.

For OS and PFS, Hazard Ratios (HRs) and their 95% Confidence Intervals (CI) were included in the meta-analysis when reported as such in the studies and estimated from Kaplan-Meier curves with the Tierney et al. approach (32) when not, with the estimations performed independently in duplicate by two reviewers to ensure consistency of the results. In case of discrepancy, another estimation was performed by a third author and if the results remained inconclusive, the estimations were not included in the meta-analysis.

Regardless of the outcome, results yielded by multivariate analyses were preferred over results yielded by univariate analyses, when available, for inclusion in the meta-analysis. When results were available on multiple ABX exposure time windows in a given study, pre-defined criteria of selection were applied to include the largest number of qualitative results and to make the most relevant analyses possible (see the complete methodology in **Supplementary Figure 1**).

### 2.5 Quality assessment

As the majority of the studies included had a retrospective design, a quality assessment was independently performed by two reviewers using the Newcastle-Ottawa scale (NOS), a star-based system that rates non-randomized studies based on the three following domains: selection of the study groups, comparability of the study groups and ascertainment of the outcomes.

### 2.6 Data analyses

#### 2.6.1 Pooled analyses

OS and PFS are respectively defined as time from immunotherapy initiation until death by any cause or loss to follow up (for OS) or until radiological evidence of progressive disease or loss to follow-up (for PFS). One of the aims of the meta-analysis was to evaluate the impact of ABX use on the survival and survival without cancer progression of cancer patients treated with ICIs by calculating pooled HRs for OS and PFS along with their 95% CI across all cohorts.

ORR and PD are treatment-related outcomes, with ORR representing the number of patients experiencing a complete or partial response, and PD equated, for the purpose of the meta-analysis, with the number of patients experiencing cancer progression. One of the aims of the meta-analysis was to assess the association between ABX use and response to treatment by calculating pooled Odds Ratios (ORs) for ORR and PD along with their 95% CI across all cohorts.

#### 2.6.2 Subgroup analyses

In order to minimize between-study heterogeneity and to determine factors influencing the impact of ABX use on survival and treatment-related outcomes, several subgroup analyses were conducted, subject to an acceptable number of cohorts per group. As the number of studies reporting data on treatment-related outcomes was relatively small, the subgroup analyses were restricted to survival outcomes.

##### 2.6.2.1 Subgroup analyses according to the cancer type

A cancer type formed a separate category if at least four cohorts of patients with that cancer type were available with data on both OS and PFS according to ABX exposure. An “Other” category was created to group cancers for which less than four cohorts of patients with that type of cancer reported data on survival outcomes, while an “Aggregated” category was defined to group cohorts having pooled patients suffering from various types of cancer.

##### 2.6.2.2 Subgroup analyses according to the antibiotic exposure time window

Five ABX exposure TWs relative to ICI initiation were selected, based on the TWs defined in the included studies and with the assumption of a stronger impact of ABX when taken around ICI initiation: [-60 days; 0], [-30 days; 0], [-60 days; 60 days], [-90 days; 120 days] and “undefined” (noted hereafter]-∞; ∞[), Day 0 being the day of initiation of the treatment with ICIs, *i.e.* the day of the first administration of immunotherapy. Of note, a patient included in the TW [-60 days; 0] may have taken ABX only in an unspecified short period included in this TW (for example, between -15 and -10 days before ICI initiation).

#### 2.6.3 Focus on non-small cell lung cancer

Lung cancers are responsible for the largest number of cancer-related deaths worldwide^2^. About 80% to 85% of all lung cancers are NSCLC. As NSCLC was one of the first cancers for which ICIs were approved^3^, it is the cancer for which the literature is the most comprehensive, with nearly half of the patients included in the meta-analysis suffering from NSCLC (and just as many studies focusing on this cancer type). For comparison, the second most represented cancer in this literature, namely UC, represents less than 15% of all patients and cohorts included. NSCLC was therefore the subject of a focus in the present meta-analysis, and some analyses were performed exclusively on the NSCLC patient population, allowing to minimize heterogeneity between studies. Thus, in addition to pooled HRs for OS and PFS and pooled ORs for ORR and PD, subgroup analyses were performed on survival outcomes according to the following ABX exposure TWs: [-60 days; 60 days], [-45 days; 45 days], [-90 days; 120 days] and]-∞; ∞[, Day 0 being the day of initiation of the treatment with ICIs. In addition, NSCLC studies were analyzed in more detail to bring out information on the baseline characteristics of NSCLC patients (histology, ECOG PS, PD-L1 expression), on the immunotherapy treatment (anti-PD-(L)1 scheme and agent, and treatment line) and on the use of ABX (ABX class, cause of prescription and route of administration).

### 2.7 Random-effect model

All calculations of HRs and ORs were performed using the inverse variance-weighted average method according to a random-effect model, to best accommodate the high heterogeneity expected from the included studies and measured using the Higgins and Thompson statistic *I* ^2^. For survival outcomes, a value of HR > 1 indicated that ABX use was negatively associated with the considered outcome, while a 95% CI > 1 indicated that the association was statistically significant. For treatment-related outcomes, a value of OR for ORR < 1 indicated that ABX use was negatively associated with treatment response, and the association was statistically significant if the 95% CI was inferior to 1. On the contrary, a value of OR for PD > 1 indicated that ABX was associated with an increased odd of cancer progression, while a 95% CI > 1 indicated that the association was statistically significant. For all analyses, a p-value ≤ 0.05 was considered to be statistically significant.

### 2.8 Publication bias and sensitivity analysis

One weakness of a meta-analysis is that it relies on the available published literature and can be affected by publication bias, which occurs when the results of a study have an impact on the decision to publish the study. For example, it is known that researchers are less likely to publish their study when their working hypothesis is not met (in our case, if antibiotics do not impact patient outcomes). Publication bias was assessed for pooled HRs for OS and PFS and pooled ORs for ORR and PD through the generation of funnel plots that were analyzed for asymmetry using Begg and Egger tests. If a publication bias was detected, its impact on the meta-analysis results was assessed *via* a trim-and-fill approach. A sensitivity analysis was also conducted to assess the risk of one individual study biasing the results using the leave-one-out approach.

All analyses were performed using R version 3.6.1 and the meta package (33, 34).

## 3 Results

### 3.1 Study selection

**Figure 2:**
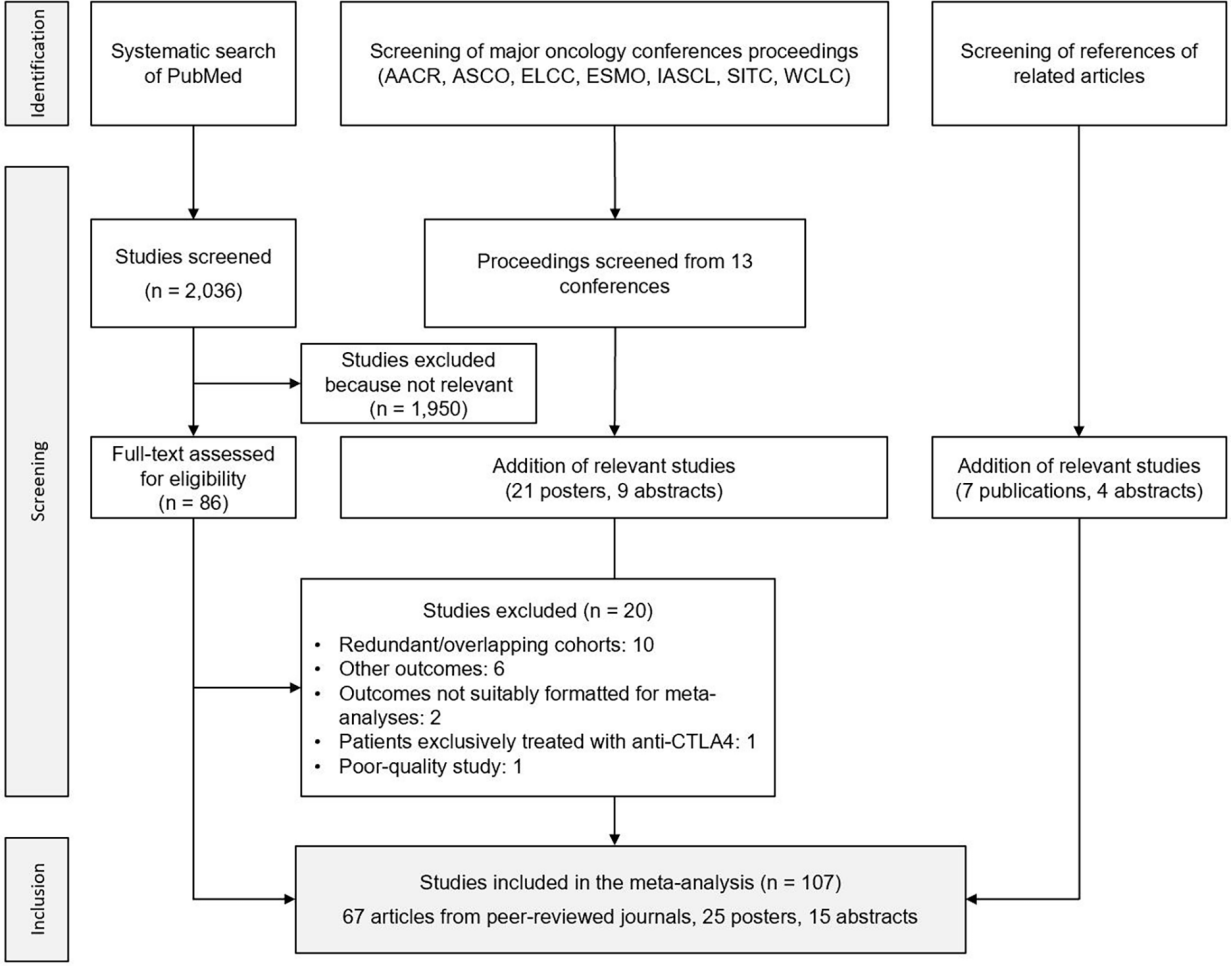
Flowchart of the search process. AACR, American Association for Cancer Research; ASCO, American Society of Clinical Oncology; ELCC, European Lung Cancer Congress; ESMO, European Society for Medical Oncology; IASCL, International Association for the Study of Lung Cancer; SITC, Society for Immunotherapy of Cancer; WCLC, World Conference on Lung Cancer.

The literature search conducted on PubMed initially retrieved 2,036 hits, of which 1,950 were excluded based on their title or abstract, leaving a total of 86 candidate studies for full-text reading. 20 studies were consequently discarded due to different reasons, including redundancy and/or overlapping cohorts, and the reporting of outcomes other than the ones retained for this meta-analysis. An additional 30 relevant studies were extracted from the screening of major oncology conference proceedings, and 11 were further identified by reviewing the references of relevant articles in the field. 67 articles published in peer-reviewed journals, 25 posters and 15 abstracts were ultimately included in the meta-analysis, representing a total of 107 articles (7, 12–14, 35–137), issued between 2017 and 2022, and reporting data on 123 independent cohorts. The results of the literature search process are displayed in **Figure 2**.

As shown in **Supplementary Table 1**, the included studies had Newcastle-Ottawa scale scores ranging from 3 to 8, with a median at 6. The missing criteria were generally item D (demonstration that outcome of interest was not present at start of study), G (adequate duration of follow-up) and H (loss to follow-up rate), and the lowest scores were mainly attributed to the abstracts. Of note, low scores do not necessarily correspond to poor-quality studies but rather to a lack of sufficient information.

### 3.2 Characteristics of studies and patients included

Baseline characteristics of studies and patients included are displayed in **Supplementary Table 2**. The very large majority of studies were retrospective analyses of patient medical records (some of which were entered into prospectively-maintained databases); only 6 studies reported prospective observational clinical trial data (13, 43, 81, 101, 110, 114).

Overall, a total of 41,663 patients diagnosed with cancer and treated with an anti-PD-(L)1-based treatment were included in the meta-analysis, among whom 11,785 (28%) were administered ABX in varying timeframes around ICI initiation.

The United States of America (USA) and Europe were the continents providing most cohorts and patients (34% of cohorts and 47% of patients for the USA, 29% of cohorts and 22% of patients for Europe), followed by Asia (22% and 9% of cohorts and patients, respectively). Within Europe, France and Spain produced most cohorts and included most patients (31% and 55% of cohorts and patients for France, respectively, and 29% and 14% for Spain).

The very large majority of patients included in the meta-analysis had a locally advanced or metastatic cancer. The number of patients enrolled in the studies ranged from 31 to 3,634, with the largest cohorts including NSCLC patients. In terms of number of patients (and of cohorts), NSCLC was by far the most represented cancer, with 40% of the 41,663 patients suffering from this cancer (and 41% of the cohorts including NSCLC patients), followed by UC (14% of patients, 12% of cohorts), melanoma (13% of patients, 7% of cohorts), renal cell carcinoma (RCC) (8% of patients, 7% of cohorts) and hepatocellular carcinoma (HCC) (4% of patients, 7% of cohorts). The remaining cohorts included patients suffering from cancer types less represented in this immuno-oncology literature, namely head and neck cancer, esophagogastric/gastric cancer, gynecologic cancers, cutaneous squamous cell carcinoma, Hodgkin lymphoma, colorectal cancer and sarcoma (each of these cancer types representing less than 3% of all patients and cohorts). Finally, 18 cohorts (16% of all patients) grouped patients suffering from various cancer types, of which NSCLC was once again the most represented cancer type (37%), followed by melanoma (29%).

The studies included were largely heterogeneous in terms of reported immunotherapy and ABX treatment characteristics, but from the review of this literature, patients seemed to be predominantly treated with anti-PD-1 monotherapy, nivolumab and pembrolizumab being the most represented ICI agents. The line of treatment greatly differed between studies, but the largest cohorts included patients receiving immunotherapy as first-line treatment for locally advanced or metastatic cancers. All studies selected varying time windows of exposure to ABX, some of them being strictly defined and very narrow around ICI initiation ([-14 days; 14 days] in Ahmed J. et al. (123)), other being broader and less defined (“after ICI initiation” in Masini et al. (131)). β-lactams and fluoroquinolones were the most used ABX in this patient population, and ABX were mostly administered *via* oral route. More detailed information on patient characteristics, anticancer treatment and antibiotic therapy is available for NSCLC patients in section 3.6.1.

### 3.3 Impact of antibiotic use on survival outcomes across all cancer types

#### 3.3.1 Global analyses

112 and 80 cohorts reported data on OS and PFS based on ABX exposure, respectively, representing 40,236 patients and 12,564 ABX users (31%) for OS and 20,318 patients and 6,223 ABX users (31%) for PFS.

**Figure 3:**
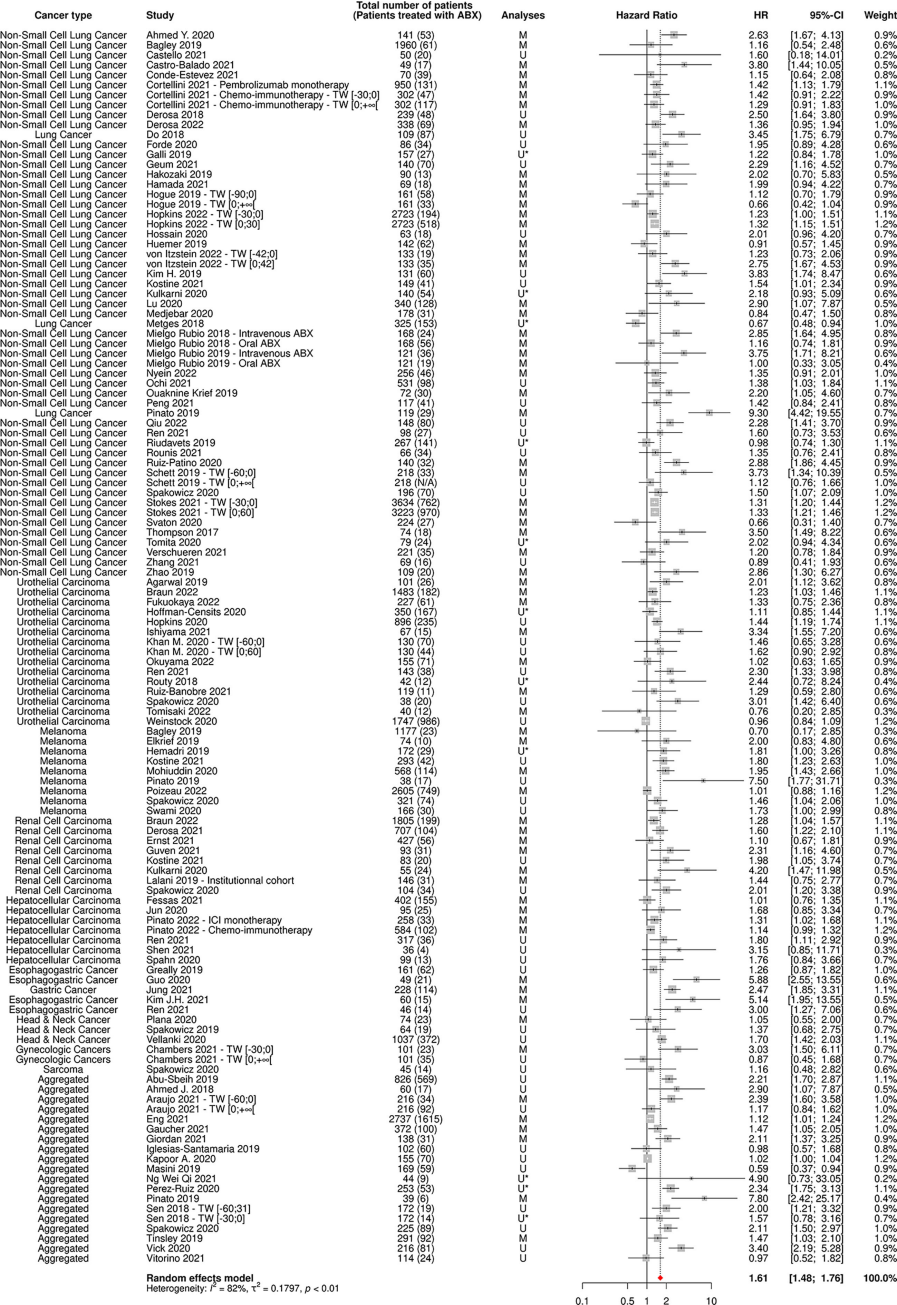
Forest plot of hazard ratios for overall survival of patients diagnosed with cancer exposed to antibiotics versus not exposed to antibiotics around immune checkpoint inhibitor treatment initiation. ABX, Antibiotic; CI, Confidence Interval; HR, Hazard Ratio; M, Multivariate; N/A, Not Available; TW, Time Window; U, Univariate; U*, Univariate, HR estimated from Kaplan-Meier curve.

**Figure 4:**
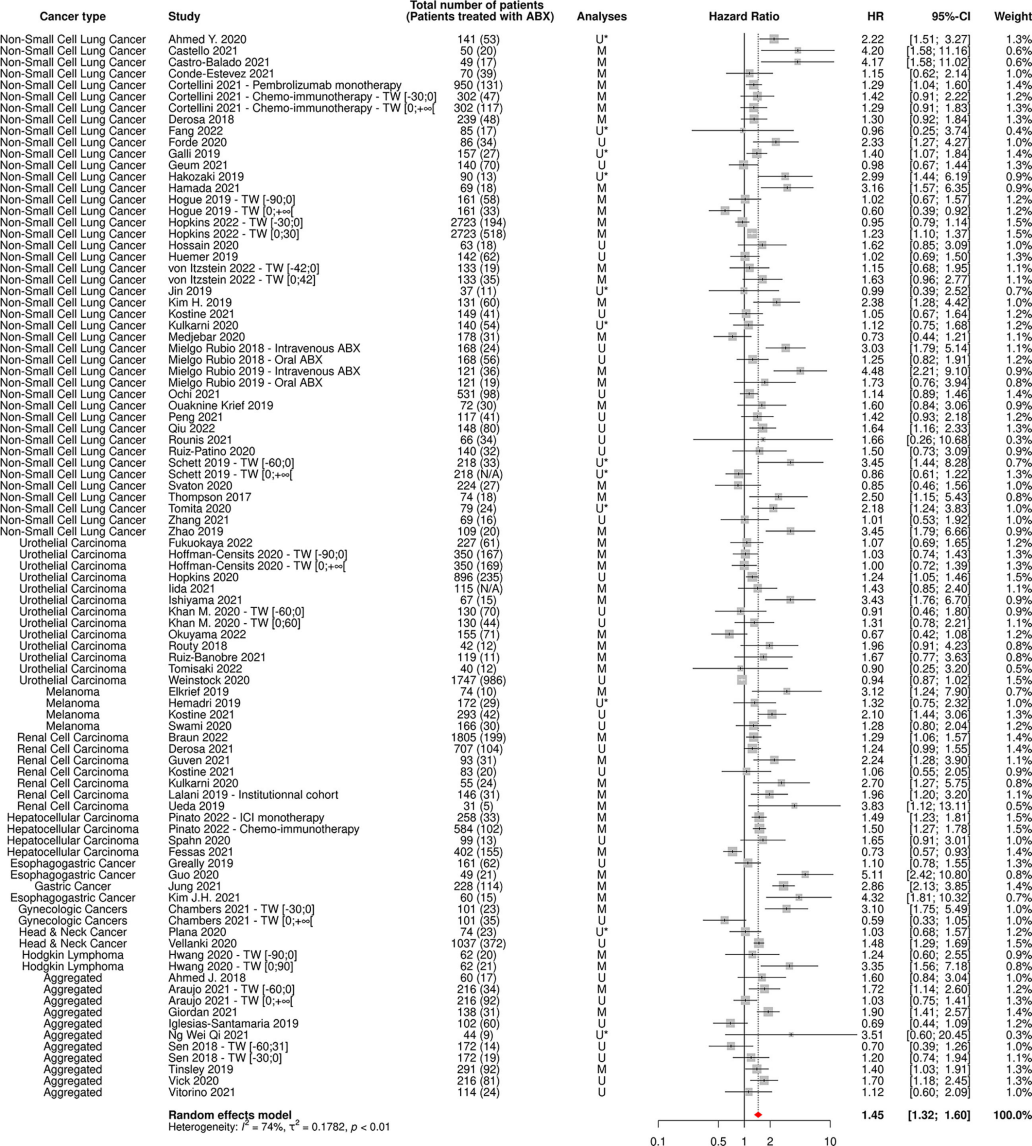
Forest plot of hazard ratios for progression-free survival of patients diagnosed with cancer exposed to antibiotics versus not exposed to antibiotics around immune checkpoint inhibitor treatment initiation. ABX, Antibiotic; CI, Confidence Interval; HR, Hazard Ratio; M, Multivariate; N/A, Not Available; TW, Time Window; U, Univariate; U*, Univariate, HR estimated from Kaplan-Meier curve.

**Figure 5:**
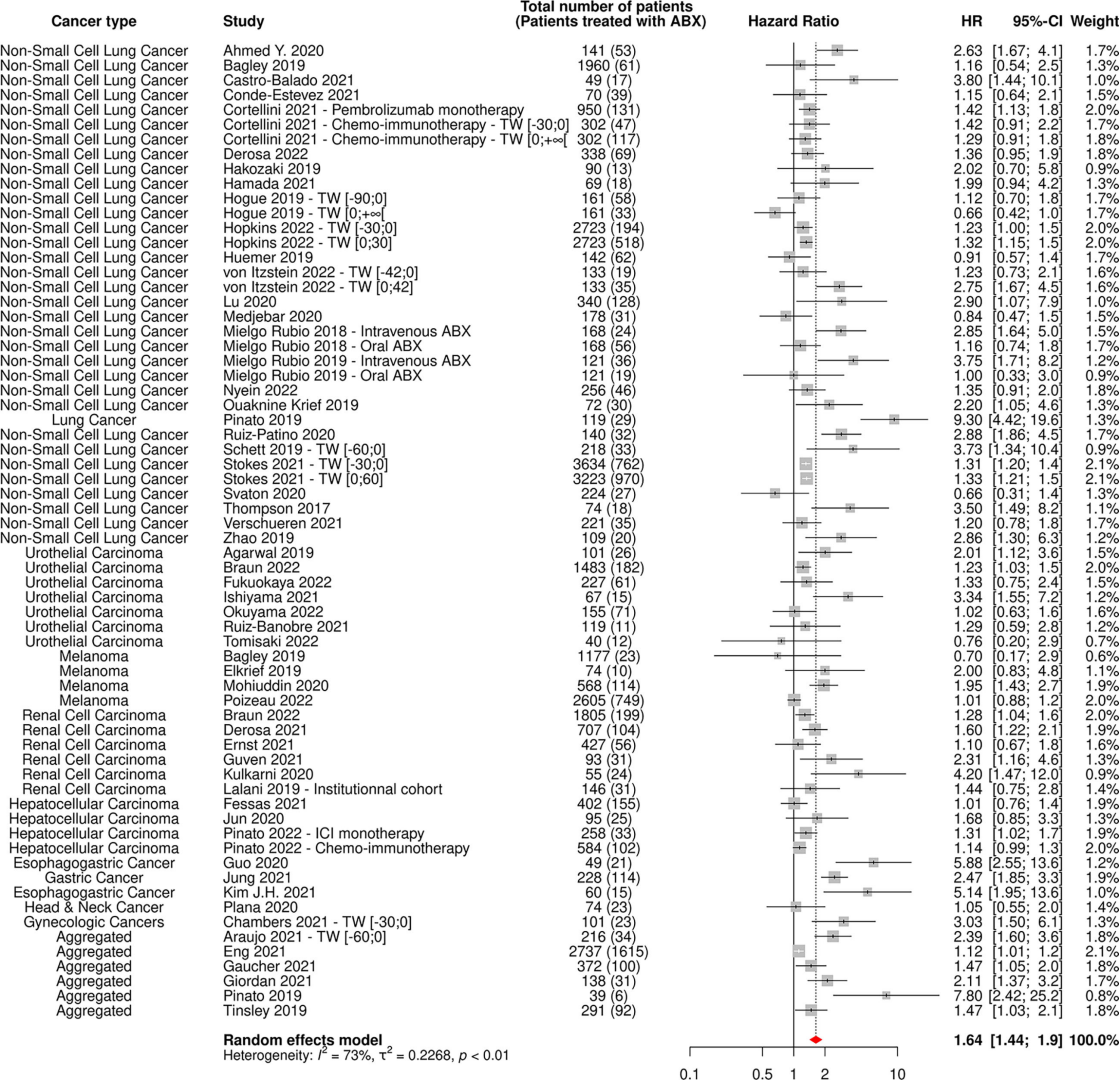
Forest plot of hazard ratios yielded from multivariate analyses for overall survival of patients diagnosed with cancer exposed to antibiotics versus not exposed to antibiotics around immune checkpoint inhibitor treatment initiation. ABX, Antibiotic; CI, Confidence Interval; HR, Hazard Ratio; TW, Time Window.

**Figure 6:**
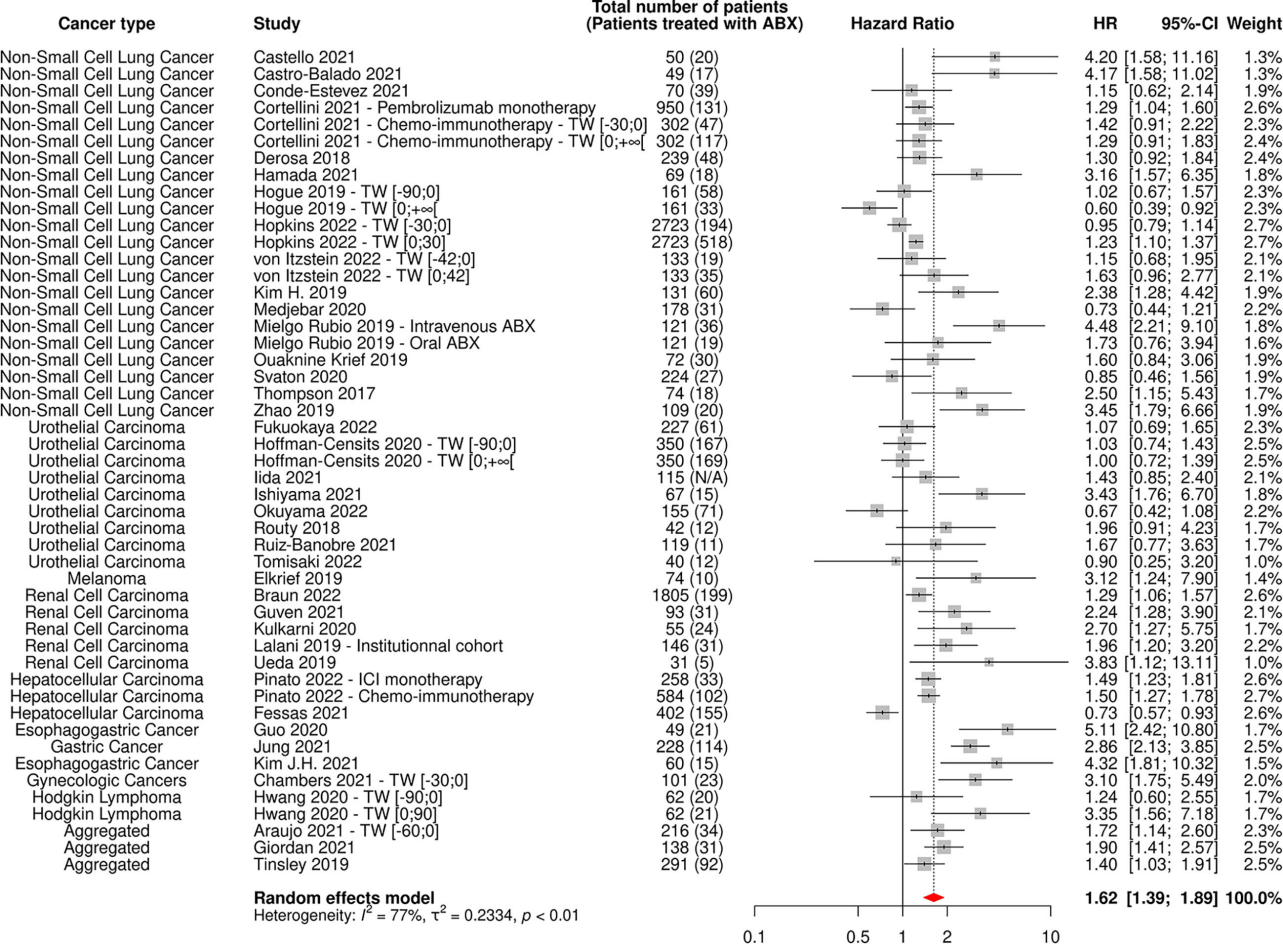
Forest plot of hazard ratios yielded from multivariate analyses for progression-free survival of patients diagnosed with cancer exposed to antibiotics versus not exposed to antibiotics around immune checkpoint inhibitor treatment initiation. ABX, Antibiotic; CI, Confidence Interval; HR, Hazard Ratio; N/A, Not Available; TW, Time Window.

The random-effect model yielded respective HRs for OS and PFS of 1.61 [95% CI 1.48-1.76] and 1.45 [95% CI 1.32-1.60] (**Figures 3**, **4**) across all cancer types and ABX exposure time windows, suggesting that ABX use is significantly associated with reduced survival and survival without progression of cancer patients treated with ICIs. When excluding HRs calculated from univariate analyses, to keep uniquely cohorts having controlled for confounding factors, the association between ABX and survival outcomes remained very highly significant, with HRs for OS and PFS being respectively of 1.64 [95% CI 1.44-1.90] and 1.62 [1.39-1.89] (**Figures 5**, **6**). Of note, the design of the study (prospective or retrospective) did not appear to have exerted an impact on the results (data not shown).

As expected, the heterogeneity factor was substantial in these global analyses (*I* ^2^ of 82% for OS, *I* ^2^ of 74% for PFS), due to the high variability observed between studies, notably in terms of type of cancer and ABX exposure time window.

#### 3.3.2 Impact of antibiotic use on survival outcomes according to the cancer type

As shown in **Table 1**, ABX were negatively associated with OS across all cancer types, and this association was particularly pronounced in NSCLC and RCC patients, with HRs for OS being of 1.60 [95% CI 1.40-1.83] and 1.65 [95% CI 1.24-2.19], respectively. ABX use was also significantly associated with a decreased PFS in patients suffering from NSCLC, RCC, and from less represented cancers. Even though ABX use was not statistically associated with a decreased PFS in patients suffering from UC, melanoma and HCC, the HRs superior to 1 and the 95% CI close to statistical significance (notably for UC and melanoma) suggest a clinically meaningful trend towards a similar negative association in these cancer types.

**Table 1:** Table of hazard ratios for overall survival and progression-free survival of patients diagnosed with cancer and exposed to antibiotics versus not exposed to antibiotics around immune checkpoint inhibitor treatment initiation, according to the cancer type.

Cancer Type	Number of Cohorts Included for OS	Pooled Number of Patients for OS (Number of ABX Users, % of ABX users)	Pooled HR OS [95% CI]	Number of Cohorts Included for PFS	Pooled Number of Patients for PFS (Number of ABX Users, % of ABX users)	Pooled HR PFS [95% CI]
**Non-Small Cell Lung Cancer**	47 cohorts *(55 HR values)*	16,163 (4,913, 30%)	1.60 [1.40-1.83]	37 cohorts *(44 HR values)*	8,421 (2,363, 28%)	1.47 [1.27-1.70]
**Urothelial Carcinoma**	14 cohorts *(15 HR values)*	5,454 (1,950, 36%)	1.45 [1.18-1.80]	11 cohorts *(13 HR values)*	3,804 (1,853, 49%)	1.18 [0.94-1.49]
**Melanoma**	9 cohorts	5,414 (1,088, 20%)	1.65 [1.16-2.34]	4 cohorts	705 (111, 16%)	1.72 [0.95-3.10]
**Renal Cell Carcinoma**	8 cohorts	3,420 (499, 15%)	1.65 [1.24-2.19]	7 cohorts	2,920 (414, 14%)	1.65 [1.14-2.38]
**Hepatocellular Carcinoma**	7 cohorts	1,791 (368, 21%)	1.35 [1.04-1.75]	4 cohorts	1,343 (303, 23%)	1.25 [0.69-2.30]
**Other Cancers**	10 cohorts *(11 HR values)*	1,865 (712, 38%)	1.92 [1.27-2.91]	8 cohorts *(10 HR values)*	1,772 (706, 40%)	1.88 [1.13-3.11]
**Aggregated**	17 cohorts *(19 HR values)*	6,129 (3,034, 50%)	1.67 [1.29-2.17]	9 cohorts *(11 HR values)*	1,353 (362, 27%)	1.28 [0.99-1.66]
**Pooled**	112 cohorts *(124 HR values)*	40,236 (12,564, 31%)	1.61 [1.48-1.76]	80 cohorts *(93 HR values)*	20,318 (6,223, 30%)	1.45 [1.32-1.60]

Statistically significant deleterious effect. Non statistically significant effect.

ABX, Antibiotic; CI, Confidence Interval; HR, Hazard Ratio; OS, Overall Survival; PFS, Progression-Free Survival.

As shown in the forest plots available in **Supplementary Figures 2, 3**, the *I* ^2^ value remained high (> 50%) for most cancer types, which was expected given the large number of factors that can induce heterogeneity, such as the diversity of histological subtypes among each cancer type and differential cancer management, for example.

#### 3.3.3 Impact of antibiotic use on survival outcomes according to the exposure time window

As shown in **Table 2**, the negative association between ABX use and survival outcomes was most pronounced when ABX were received in the one or two months preceding or following the initiation of immunotherapy, with the HR for OS reaching the high value of 2.24 [95% CI 1.66-3.03] in the [-30 days; 0] TW. It appears that the TW of ABX exposure relative to the date of initiation of the ICI treatment has an impact on the observed clinical outcomes, with ABX taken long before or after the initiation of the ICI initiation having a less pronounced impact on patient outcomes, compared with ABX taken just before or just after ICI initiation.

**Table 2:** Table of hazard ratios for overall survival and progression-free survival of patients diagnosed with cancer and exposed to antibiotics versus not exposed to antibiotics around immune checkpoint inhibitor treatment initiation, according to the antibiotic exposure time window.

Time Window of Exposure to ABX in Relation to ICI Treatment Initiation (Days)	Number of Cohorts Included for OS	Pooled Number of Patients for OS (Number of ABX users, % of ABX users)	Pooled HR OS [95% CI]	Number of Cohorts Included for PFS	Pooled Number of Patients for PFS (Number of ABX users, % of ABX users)	Pooled HR PFS [95% CI]
[-60; 0]	14 cohorts	5,055 (1,003, 20%)	1.72 [1.36-2.18]	10 cohorts	1,457 (333, 23%)	1.60 [1.14-2.23]
[-30; 0]	19 cohorts	9,539 (1,599, 17%)	2.24 [1.66-3.03]	14 cohorts	5,364 (658, 12%)	1.77 [1.33-2.35]
[-60; 60]	61 cohorts *(63 HR values)*	21,855 (5,009, 23%)	1.68 [1.53-1.85]	43 cohorts *(45 HR values)*	12,705 (3,264, 26%)	1.59 [1.39-1.82]
[-90; 120]	9 cohorts	4,139 (1,235, 30%)	1.26 [1.02-1.57]	9 cohorts *(10 HR values)*	1,113 (430, 39%)	1.34 [1.02-1.76]
]-∞; ∞[	19 cohorts	7,000 (2,007, 29%)	1.33 [1.03-1.73]	14 cohorts	4,185 (959, 23%)	1.02 [0.84-1.24]

Statistically significant deleterious effect. Non statistically significant effect.

ABX, Antibiotic; CI, Confidence Interval; HR, Hazard Ratio; ICI, Immune Checkpoint Inhibitor; OS, Overall Survival; PFS, Progression-Free Survival.

As shown in the forest plots available in **Supplementary Figures 4, 5**, heterogeneity remained high (*I* ^2^ > 50%) for most TWs.

### 3.4 Impact of antibiotic use on treatment-related outcomes across all cancer types

44 and 38 cohorts reported data on ORR and PD based on ABX exposure, respectively, representing 7,854 patients and 1,997 ABX users (25%) for ORR and 6,142 patients and 1,654 ABX users (27%) for PD.

**Figure 7:**
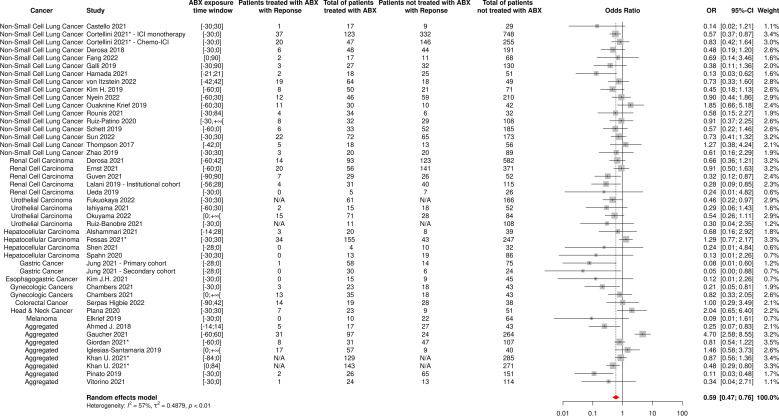
Forest plot of odds ratios of the overall response rate of patients diagnosed with cancer and exposed to antibiotics versus not exposed to antibiotics around immune checkpoint inhibitor treatment initiation. ABX, Antibiotic; CI, Confidence Interval; OR, Odds Ratio; Response, Complete or Partial Response. *The OR value from multivariate analyses was available for this study and therefore used as such in the meta-analysis.

**Figure 8:**
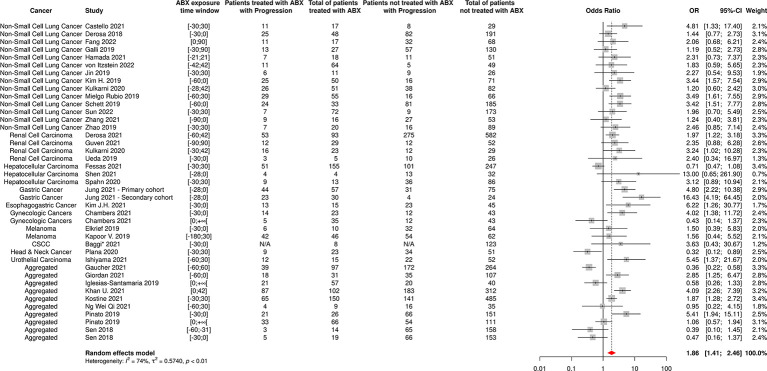
Forest plot of odds ratios of the progressive disease rate of patients diagnosed with cancer and exposed to antibiotics versus not exposed to antibiotics around immune checkpoint inhibitor treatment initiation. ABX, Antibiotic; CI, Confidence Interval; CSCC, Cutaneous Squamous Cell Carcinoma; OR, Odds Ratio; Progression, Cancer progression. *The OR value from multivariate analyses was available for this study and therefore used as such in the meta-analysis.

The random-effect model yielded ORs for ORR and PD of 0.59 [95% CI 0.47-0.76] and 1.86 [95% CI 1.41-2.46], respectively (**Figures 7**, **8**), suggesting that ABX use was significantly and negatively associated with impaired response to treatment among cancer patients receiving ABX, with both a reduced odd of response and an increased odd of cancer progression among ABX users.

As expected, the heterogeneity factor was substantial (*I* ^2^ of 57% for ORR, *I* ^2^ of 74% for PD) in these analyses.

### 3.5 Publication bias and sensitivity analysis

Funnel plots for OS, PFS, ORR and PD are available in **Supplementary Figures 6–9**. Begg and/or Egger tests indicate the existence of publication bias, as suggested by asymmetrical funnel plots, in global analyses associating ABX use with OS, PFS, ORR and PD (OS: p-value for Begg test: 0.7280, p-value for Egger test < 0.0001; PFS: p-value for Begg test: 0.0042, p-value for Egger test < 0.0001; ORR: p-value for Begg test: 0.0012, p-value for Egger test: 0.0014; PD: p-value for Begg test: 0.6212, p-value for Egger test: 0.0509). However, the trim-and-fill approach implemented indicated that the publication bias was unable to significantly affect the results for OS, PFS and PD, and that antibiotic use remained significantly associated with decreased OS (HR 1.44 [95% CI 1.30-1.59]) and PFS (HR 1.38 [95% CI 1.24-1.54]), and increased PD (OR 1.58 [95% CI 1.18-2.12]). On the contrary, the suggested deleterious impact of ABX treatment on the ORR did not remain statistically significant (OR 0.78 [95% CI 0.59-1.03]), although a clear trend for an impaired response still persisted. Besides, the sensitivity analysis performed using the leave-one-out-approach demonstrated that no single study was able to significantly influence the pooled HRs for OS and PFS, as well as the pooled ORs for ORR and PD (data not shown), supporting the reliability of the results.

### 3.6 Impact of antibiotic use on NSCLC patient clinical outcomes

#### 3.6.1 Characteristics of NSCLC patients, immunotherapy and antibiotic treatment

A total of 50 independent cohorts including 16,529 patients (46% in the USA, 22% in Europe) suffering from NSCLC were included in the meta-analysis, of whom 5,022 (30%) were given ABX in the three months prior to ICI initiation and/or during immunotherapy.

The reported data on NSCLC patient characteristics, anticancer treatment and antibiotic therapy were largely heterogeneous between studies.

Pooling the 38 NSCLC cohorts reporting histologic data (10,561 patients), non-squamous cell carcinoma and squamous cell carcinoma accounted for 62% and 17% of histological subtypes, respectively. According to the 32 cohorts reporting performance status scores (6,323 patients), 87% of NSCLC patients had an ECOG PS equal to 0 or 1, with one-third of these patients having an ECOG PS of 0 and two-thirds having an ECOG PS of 1. Regarding expression of PD-L1 protein at tumor cell surface, as expressed by the Tumor Proportion Score (TPS), a TPS ≥ 50% was the most represented PD-L1 expression level among NSCLC patients, accounting for 45% of the 4,413 patients included in the 20 cohorts reporting such data, corresponding to an over-representation of this level of PD-L1 expression compared to the 30% rate usually observed (138, 139).

Among the 38 cohorts documenting treatments in more detail (representing 6,652 patients), the vast majority of patients (90%) received an anti-PD-(L)1-based treatment as monotherapy. Nivolumab, pembrolizumab (both anti-PD-1 agents) and atezolizumab (anti-PD-L1) respectively accounted for 40%, 31% and 28% of the molecules received (reported in 31 cohorts for 10,728 patients). 70% of patients were treated with anti-PD-(L)1-based treatments as first-line (22 cohorts, 5,651 patients).

β-lactams, fluoroquinolones and macrolides were the most represented classes used by NSCLC patients, accounting respectively for 52%, 27% and 14% of ABX prescriptions within the 23 cohorts documenting ABX use (1,531 ABX prescriptions). This was not unexpected considering the relatively broad spectrum of antimicrobial activity of these ABX classes, which are often used for oncology patients. In the 19 cohorts reporting the indication for ABX use (917 prescriptions), more than half (51%) of the prescriptions were indicated to treat respiratory tract infections including suspected pneumonia. Finally, the oral route was the most represented route of administration and accounted for 66% of the 537 prescriptions documented in 12 cohorts, which was expected as most of these patients are treated in the community setting.

#### 3.6.2 Impact of antibiotic use on clinical outcomes of NSCLC patients

As previously mentioned, ABX use was significantly associated with impaired OS and PFS of NSCLC patients, as reported by the HRs respectively measured at 1.60 [95% CI 1.40-1.83] and 1.47 [95% CI 1.27-1.70] (**Table 1** and **Supplementary Figures 2, 3**).

Similarly to the results obtained in the global analyses grouping all cancer types, excluding studies reporting only univariate analyses did not substantially change the results, with HRs being of 1.62 [95% CI 1.34-2.0] for OS and 1.51 [95% CI 1.18-1.93] for PFS, respectively (**Supplementary Figures 10, 11**). Of note, the most examined potential confounding factors for OS were, in this order, ECOG PS, age, sex, treatment line, smoking status/history, histology, other co-medications, cancer stage at diagnosis and presence of central nervous system metastases. The factors were broadly the same for PFS. Among the potential confounding factors, ECOG PS, histology and use of other co-medications were the factors with the greatest impact on OS and PFS (data not shown).

**Figure 9:**
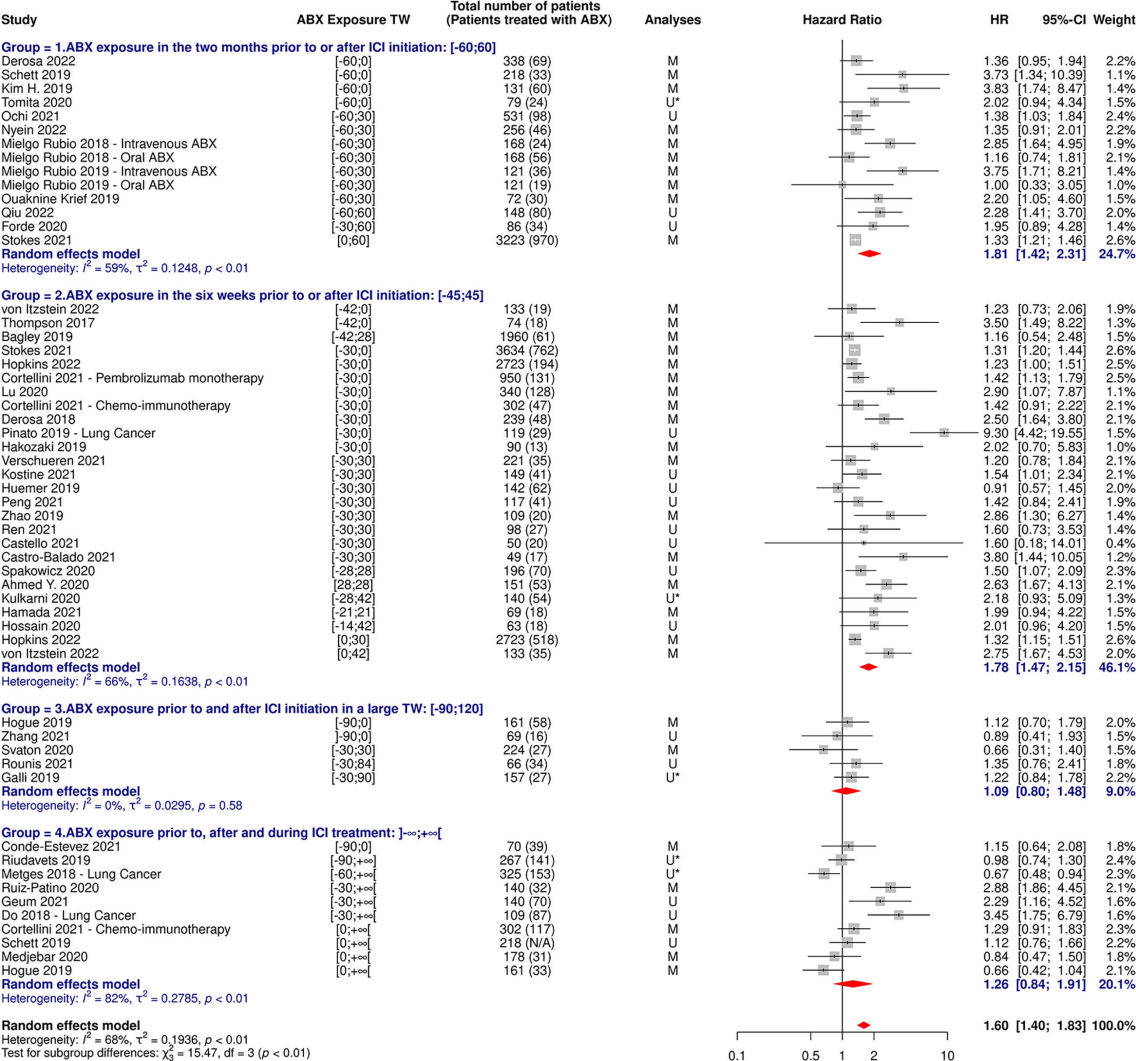
Forest plot of hazard ratios for overall survival of patients diagnosed with non-small cell lung cancer and exposed to antibiotics versus not exposed to antibiotics around immune checkpoint inhibitor treatment initiation, according to the antibiotic exposure time window. ABX, Antibiotic; CI, Confidence Interval; HR, Hazard Ratio; M, Multivariate; N/A, Not Available; TW, Time Window; U, Univariate; U*, Univariate, HR estimated from Kaplan-Meier curve.

**Figure 10:**
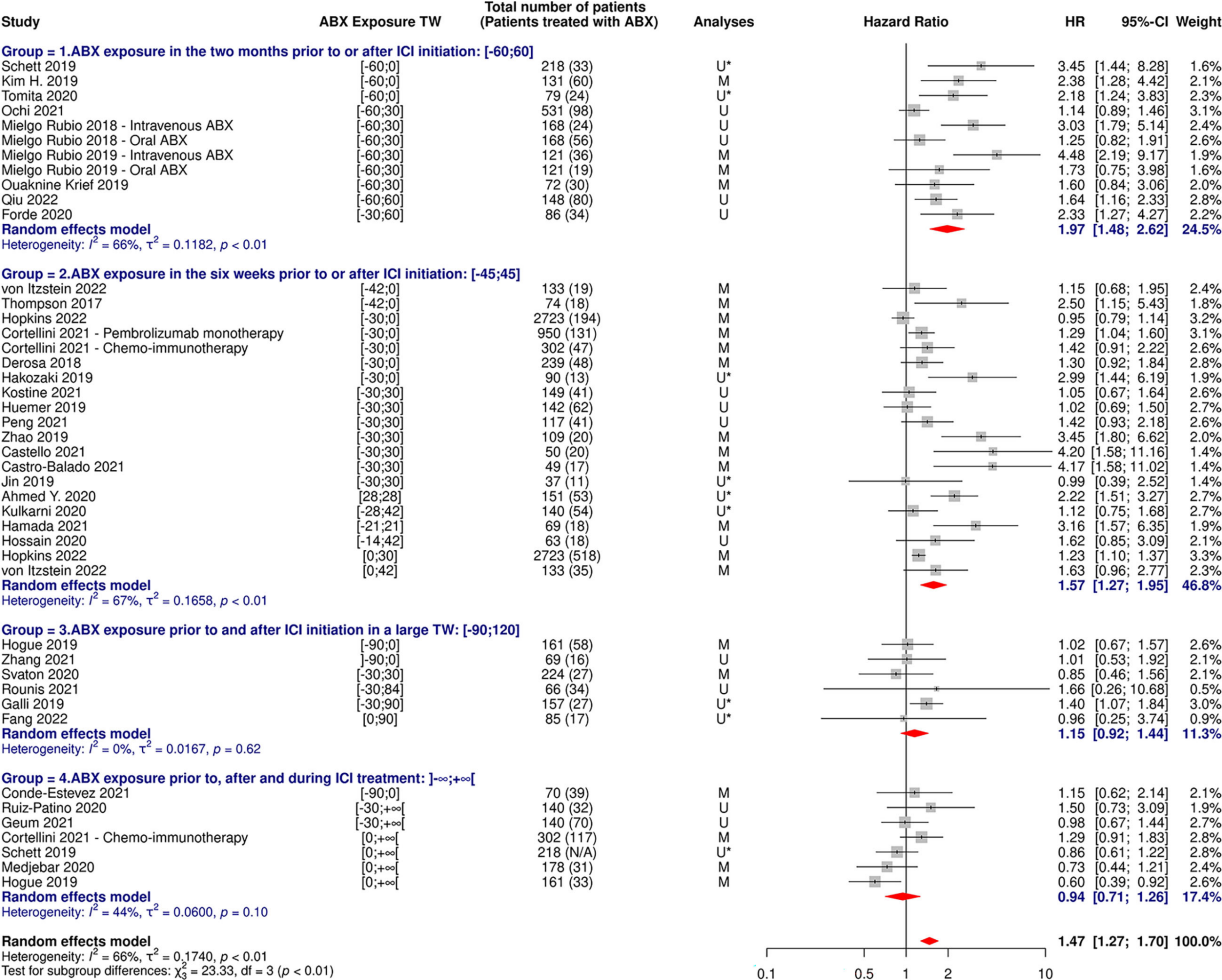
Forest plot of hazard ratios for progression-free survival of patients diagnosed with non-small cell lung cancer and exposed to antibiotics versus not exposed to antibiotics around immune checkpoint inhibitor treatment initiation, according to the antibiotic exposure time window. ABX, Antibiotic; CI, Confidence Interval; HR, Hazard Ratio; M, Multivariate; N/A, Not Available; TW, Time Window; U, Univariate; U*, Univariate, HR estimated from Kaplan-Meier curve.

As shown in **Table 3** and **Figures 9**, **10**, OS and PFS were particularly reduced in patients treated with ABX within the weeks preceding or following ICI initiation, whereas the suggested damaging impact was not statistically significant when ABX were taken in timeframes more distant to immunotherapy start.

**Table 3:** Table of hazard ratios for overall survival and progression-free survival of patients diagnosed with non-small cell lung cancer and exposed to antibiotics versus not exposed to antibiotics around immune checkpoint inhibitor treatment initiation, according to the antibiotic exposure time window.

Time Window of Exposure to ABX in Relation to ICI Treatment Initiation (Days)	Number of Cohorts Included for OS	Pooled Number of Patients for OS (Number of ABX users, % of ABX users)	Pooled HR OS [95% CI]	Number of Cohorts Included for PFS	Pooled Number of Patients for PFS (Number of ABX users, % of ABX users)	Pooled HR PFS [95% CI]
[-60; 60]	12 cohorts *(14 HR values)*	5,372 (1,579, 29%)	1.81 [1.42-2.31]	9 cohorts *(11 HR values)*	1,554 (494, 32%)	1.97 [1.48-2.62]
[-45; 45]	23 cohorts *(26 HR values)*	12,286 (2,500, 20%)	1.78 [1.47-2.15]	18 cohorts *(20 HR values)*	5,577 (1,368, 25%)	1.57 [1.27-1.95]
[-90; 120]	5 cohorts	677 (162, 24%)	1.09 [0.80-1.48]	6 cohorts	762 (179, 23%)	1.15 [0.92-1.44]
]-∞; ∞[	10 cohorts	1,910 (703, >37%)	1.26 [0.84-1.91]	7 cohorts	1,209 (322, 27%)	0.94 [0.71-1.26]

Statistically significant deleterious effect. Non statistically significant effect.

ABX, Antibiotic; CI, Confidence Interval; HR, Hazard Ratio; ICI, Immune Checkpoint Inhibitor; OS, Overall Survival; PFS, Progression-Free Survival.

**Figure 11:**
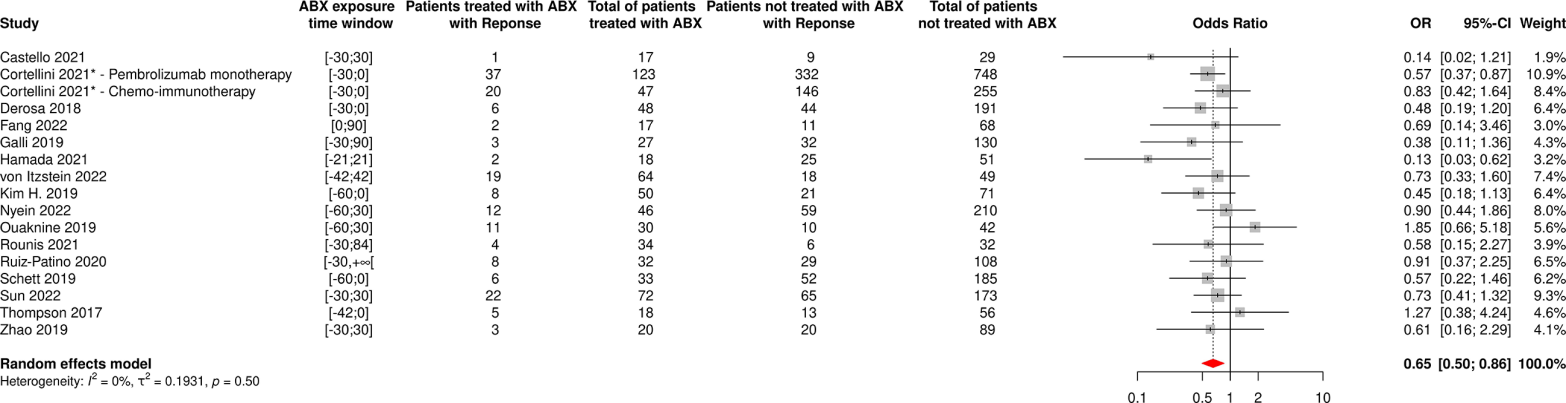
Forest plot of odds ratios of the overall response rate of patients diagnosed with non-small cell lung cancer and exposed to antibiotics versus not exposed to antibiotics around immune checkpoint inhibitor treatment initiation. ABX, Antibiotic; CI, Confidence Interval; OR, Odds Ratio; Response, Complete or Partial Response. *The OR value from multivariate analyses was available for this study and therefore used as such in the meta-analysis.

**Figure 12:**
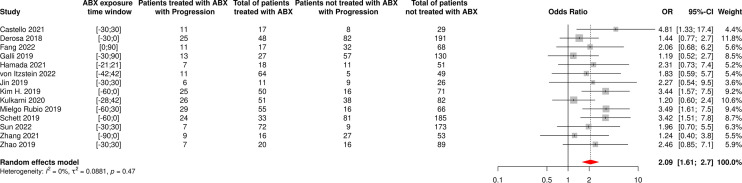
Forest plot of odds ratios of the progressive disease rate of patients diagnosed with non-small cell lung cancer and exposed to antibiotics versus not exposed to antibiotics around immune checkpoint inhibitor treatment initiation. ABX, Antibiotic; CI, Confidence Interval; OR, Odds Ratio; Progression, Cancer progression.

17 and 14 cohorts reported data on ORR and PD based on ABX exposure, respectively, representing 3,296 NSCLC patients and 696 ABX users (21%) for ORR and 1,803 NSCLC patients and 499 ABX users (28%) for PD. The random-effect models yielded ORs for ORR and PD of 0.65 [95% CI 0.50-0.86] and 2.09 [95% CI 1.61-2.70], respectively, confirming significantly impaired response to treatment among NSCLC patients having received ABX around ICI initiation (**Figures 11**, **12**).

#### 3.6.3 Publication bias and sensitivity analysis

Funnel plots for OS, PFS, ORR and PD are available in **Supplementary Figures 12–15** and suggested, again, some level of asymmetry. Begg and Egger tests both suggested the existence of publication bias in global analyses associating ABX use with survival outcomes (OS: p-value for Begg test: 0.0062, p-value for Egger test: 0.0047; PFS: p-value for Begg test: 0.0020, p-value for Egger test: 0.0037), but not in global analyses associating ABX use and treatment-related outcomes (ORR: p-value for Begg test: 0.2165, p-value for Egger test: 0.3866; PD: p-value for Begg test: 0.7016, p-value for Egger test: 0.3909). The trim-and-fill approach implemented indicated that such publication bias was unable to significantly affect the results for OS and PFS, with HRs being respectively re-calculated at 1.43 [95% CI 1.23-1.67] and 1.40 [95% CI 1.20-1.64]. In addition, the sensitivity analysis performed using the leave-one-out-approach demonstrated for all four outcomes that no single study was able to significantly influence the results, validating their reliability (data not shown).

## 4 Discussion

With the increasing use of immune checkpoint inhibitors in cancer care, considerable efforts have been made to identify factors that may alter their effectiveness, and ABX use has recently emerged as one of them, as demonstrated by numerous retrospective and prospective studies (7, 12–14, 35–137) and several meta-analyses (15–30) published on the topic. Our meta-analysis stands out from the others in that it included more than three-fold the number of patients compared with the most comprehensive published meta-analysis so far (23), allowing to perform reliable subgroup analyses evaluating the potential differential association of ABX use with outcomes depending on the cancer type and on the ABX exposure time window. The numbers of cohorts and patients included in our meta-analysis were also sufficient to explore the impact of ABX use on short-term treatment-related outcomes, namely ORR and PD, which has been relatively understudied to date. Response-based endpoints, such as ORR and PD, although investigator-assessed, are likely to be less affected by the patient inherent state of health, or subsequent lines of therapy, than overall survival outcome. Furthermore, such outcomes closely reflect the anti-tumor effect of the treatment (shrinkage *versus* escape *versus* growth of the tumor). Demonstrating a deleterious impact of antibiotics on response-based endpoints could therefore be an interesting way to dispose of possible confounding factors (such as the occurrence of a severe infection requiring an antibiotic treatment), that may be associated with a poorer prognosis without being directly related to the impact of ABX use on the gut microbiome. For all these reasons, our analyses provide some novel insights that may be useful in clarifying the specific settings in which ABX should be prescribed in cancer patients treated with ICIs.

Using a random-effect model, we firstly demonstrated that ABX use was associated with impaired survival outcomes in the entire cancer patient population receiving ICIs, which was subsequently confirmed by the analyses of publication bias and sensitivity, that confirmed the reliability and the robustness of the results, and which is in accordance with the meta-analyses previously published on the subject (15–30). Exclusion of cohorts not having performed multivariate analyses further showed that this suggested deleterious impact persisted despite adjustment for confounding factors, suggesting that ABX use is an independent predictor factor for OS and PFS. The negative association of ABX and OS held across all cancer types investigated, namely NSCLC, UC, melanoma, RCC and HCC, with the strongest effects observed in NSCLC and RCC patients. However, the association with PFS was not significant in melanoma, UC and HCC patients (although close to statistical significance, and clinically meaningful for melanoma and UC). These differential effects are likely explained in part by the fewer numbers of cohorts included in each category for PFS, but it also could be caused by heterogeneity between cancers and patients as well as different modalities of ABX use. NSCLC patients are, for example, particularly prone to lung infections due to smoking that impairs local epithelial immunity and cilia-induced mucus clearance (140). Nevertheless, the publication of more and more articles showing a negative association between ABX and outcomes in more and more types of cancer, in patients not specifically affected by respiratory infections, seems to suggest a common effect to a large part of cancer types. The deleterious impact of ABX did not seem to vary according to the route of administration, suggesting that it is not related to the severity of the underlying infection. Strikingly, ABX were strongly associated with decreased survival outcomes when taken in the few weeks prior to or following ICI initiation for patients suffering from all types of cancer and especially for NSCLC patients. The association between ABX use, OS and PFS seems to depend from the TW of ABX exposure relative to the date of initiation of the ICI treatment: ABX taken long before or after ICI start have a less pronounced impact on patient outcomes, compared with ABX taken just before or just after ICI initiation. This result supports the hypothesis of an involvement of the gut microbiome, as patients having received ABX near ICI initiation probably have a highly dysbiotic microbiome at the time of starting ICI. Finally, ABX were negatively associated to treatment-related outcomes, with a decreased odd of response and an increased odd of cancer progression in patients suffering from all types of cancer and notably in NSCLC patients. These results remained significant following publication bias and sensitivity analyses, except for the OR for ORR of patients diagnosed with any type of cancer (although a clear trend for an impaired response persisted), confirming that ABX are also negatively associated with the response to ICI treatment. ABX prophylaxis is now recommended in cancer patients receiving chemotherapy who are at high risk of grade 4 neutropenia and sepsis, and for whom the standard of care is now concomitant chemotherapy and ICIs. The results of this meta-analysis plead for caution in using such routine ABX prophylaxis when ICIs are considered. However, our analysis included a minority of studies dedicated to chemo-immunotherapy treatment and the indication for prophylactic ABX should be balanced with the risk of life-threatening neutropenia, taking into account individual characteristics (age, comorbidities, previous grade 4 neutropenia events, *etc.*) (141).

This systematic review and meta-analysis work certainly cannot discuss causality between ABX use and impaired clinical outcomes of cancer patients treated with ICIs, nor can it elucidate the underlying mechanisms involved. It can only show an association between ABX use and reduced ICI efficacy, and growing evidence in the literature and in the clinic suggest an involvement of the intestinal microbiome and ABX-induced dysbiosis. A high gut microbiome diversity at baseline was for example significantly associated with favorable clinical outcomes in several studies on NSCLC and melanoma patients (49, 56, 142). Our team recently demonstrated that FMT from ABX-treated healthy volunteers into germ-free mice altered the response of tumor-bearing mice to anti-PD-1 treatment, whereas FMT from healthy individuals having received both ABX and an ABX-adsorbent delivered to the colon that acted to protect the intestinal microbiome against dysbiosis was able to preserve ICI efficacy in the same mouse model (143). Besides, two recent clinical trials conducted in patients whose metastatic melanoma was refractory to a previous treatment with anti PD-(L)1 monoclonal antibodies suggested that FMT from other patients whose cancer responded to the same immunotherapy enabled to overcome the resistance of their tumor to PD-(L)1 blockade (144, 145). The mechanisms by which the gut microbiome impacts response to immunotherapy remain largely debated, but two types of non-mutually exclusive conjectures are being discussed: an adjuvant effect and non-antigen specific improvement of the anti-tumor response by an increased “immune tonus” on one hand (146), and an antigenic effect with improvement of anti-tumor immune response by antigenic mimicry and cross reactivity with phage or bacterial encoded antigens, on the other hand (147). Interestingly, the damaging impact of ABX on the clinical outcomes of cancer patients treated with ICIs, that remains to be proved, could also be exerted on the outcomes of patients treated with other types of cancer immunotherapy. In a recent retrospective study including 228 patients suffering from hematological cancers and treated with Chimeric Antigenic Receptor – T cells (CAR-T) therapy, ABX use in the four weeks preceding treatment initiation was indeed associated to worse survival and increased neurotoxicity (148). In another retrospective study presentation at ESMO 2022, ABX use in the three weeks prior to CAR-T therapy initiation was also associated to impaired survival outcomes and increased cancer progression (149). Changes in the composition of the gut microbiome was also associated to clinical outcomes. The intestinal microbiome, through its complex interplay with the immune system, could therefore be crucial for response to cancer immunotherapy in most cancers, making personalized patient management and microbiome research essential.

Several inherent limitations to our meta-analysis are worth mentioning. First, a meta-analysis depends in part on the studies included, and most of them, in this case, were retrospective and therefore heterogeneous and incomplete in terms of reported data. Heterogeneity was very high in most of our analyses, although we attempted to mitigate it by performing subgroup analyses. Besides, the potential differential impact of ABX use could not be evaluated according to patient and treatment baseline characteristics such as PD-L1 expression or line of treatment, due to the lack of cohorts having reported such data, whereas these factors might have been of importance. Similarly, too few studies reported detailed data according to ABX treatment characteristics (duration of use, ABX class and route of administration) on patient outcomes, thus making it impossible to refine results in this regard. Further research in the field shall investigate the differential impact of ABX classes or treatment schemes. Besides, some TWs may have been overlapping without the authors’ knowledge. For example, a patient exposed to ABX in the 30 days prior to ICI initiation could have received ABX in the 30 days following treatment start and only be included in the first category. In addition, the retrospective design made it impossible to characterize the microbiome of patients before and during ICI treatment. Second, statistical analyses demonstrated the existence of publication bias within the literature, which we attempted to mitigate by including unpublished studies such as conference proceedings abstracts and by performing analyses that confirmed that publication bias could not affect most of our results. Third, the studies have included patients whose cancer characteristics and immunotherapy treatment are no longer the most representative of the real-world setting. Indeed, studies mainly included patients treated with ICI as single agent, which no longer corresponds to standard of care, for most oncology indications, as ICIs are now mainly given in combination with chemotherapy or other treatment modalities. The impact of ABX use on patients treated with such combinations deserves to be further investigated, as only a few articles have investigated this matter (41, 52, 82) and do not allow to draw clear conclusions. Besides, nivolumab was the most represented ICI agent used in the papers included in this meta-analysis, whereas it has been largely supplanted by pembrolizumab in clinical practice since 2017. There was also an over-representation of high PD-L1 expressors (PD-L1 expression ≥ 50%) in the cohorts included in the meta-analysis compared to the real-world setting in link with the large number of single ICI agent studies. Fourth, ABX intake could not be the cause of worse outcomes but simply a marker of a degraded state in a patient, even though the performance of multivariate analyses precisely aims at adjusting for patient baseline characteristics. Finally, other medical interventions (*e.g.* prior radiotherapy), patient care and other co-medications besides ABX, such as proton pomp inhibitors and steroids, may also play a role in modulating ICI efficacy, and were not necessarily captured in the included studies. A meta-analysis evaluating the impact of proton pump inhibitor use on the clinical outcomes of 15,957 cancer patients treated with ICIs effectively concluded that their usage was negatively associated with survival outcomes (150). A negative association between steroid use and survival outcomes was also reported in another meta-analysis including 4,045 cancer patients receiving ICIs, suggesting the value of further studying the role of other co-medications (151).

In summary, this study demonstrated that ABX use around ICI initiation was negatively associated to survival and treatment-related outcomes of cancer patients, particularly when ABX were taken shortly before or after ICI start, suggesting that ABX prescription should be cautiously considered in cancer patients receiving an anti-PD-(L)1-based treatment. Future larger, prospective observational, multicentric studies evaluating changes of the intestinal microbiome and patient outcomes during immunotherapy, and interventional, controlled, randomized trials involving microbiome modifiers such as FMT or microbiome protectors, are crucially needed to explore the hypothesis of an involvement of the microbiome, elucidate the mechanisms at stake and restore the effectiveness of immunotherapies to improve patient care. It is only through such studies, which will put an end to the current publication bias by allowing analyses on more homogeneous populations, that we will be able to definitively conclude whether or not antibiotics have a deleterious impact on the clinical outcomes of cancer patients, and take the appropriate measures to improve the treatment of these patients.

## Data availability statement

The original contributions presented in the study are included in the article/**Supplementary Material**. Further inquiries can be directed to the corresponding author.

## Author contributions

AC, CL, JC, and P-AB designed the study, ran the systematic search, collected the data, and performed the analyses. AC, CL, JG, FV, GZ, JC, and P-AB discussed the results and critically reviewed the manuscript. All authors contributed to the article and approved the submitted version.

## Footnotes

1 Cancer Research Institute. https://www.cancerresearch.org/scientists/immuno-oncology-landscape/pd-1-pd-l1-landscape

2 Global Cancer Observatory. https://gco.iarc.fr/

3 Cancer Research Institute. https://www.cancerresearch.org/scientists/immuno-oncology-landscape/pd-1-pd-l1-landscape
