# Supplementary material for: A systematic review and meta-analysis evaluating the impact of antibiotic use on the clinical outcomes of cancer patients treated with immune checkpoint inhibitors
Source: Front Oncol. 2023 Mar 2;13:1075593. doi: 10.3389/fonc.2023.1075593 (PMC10019357; doi:10.3389/fonc.2023.1075593)
Supplement: Supplementary file 1 [file DataSheet_1.docx]

Supplementary Material


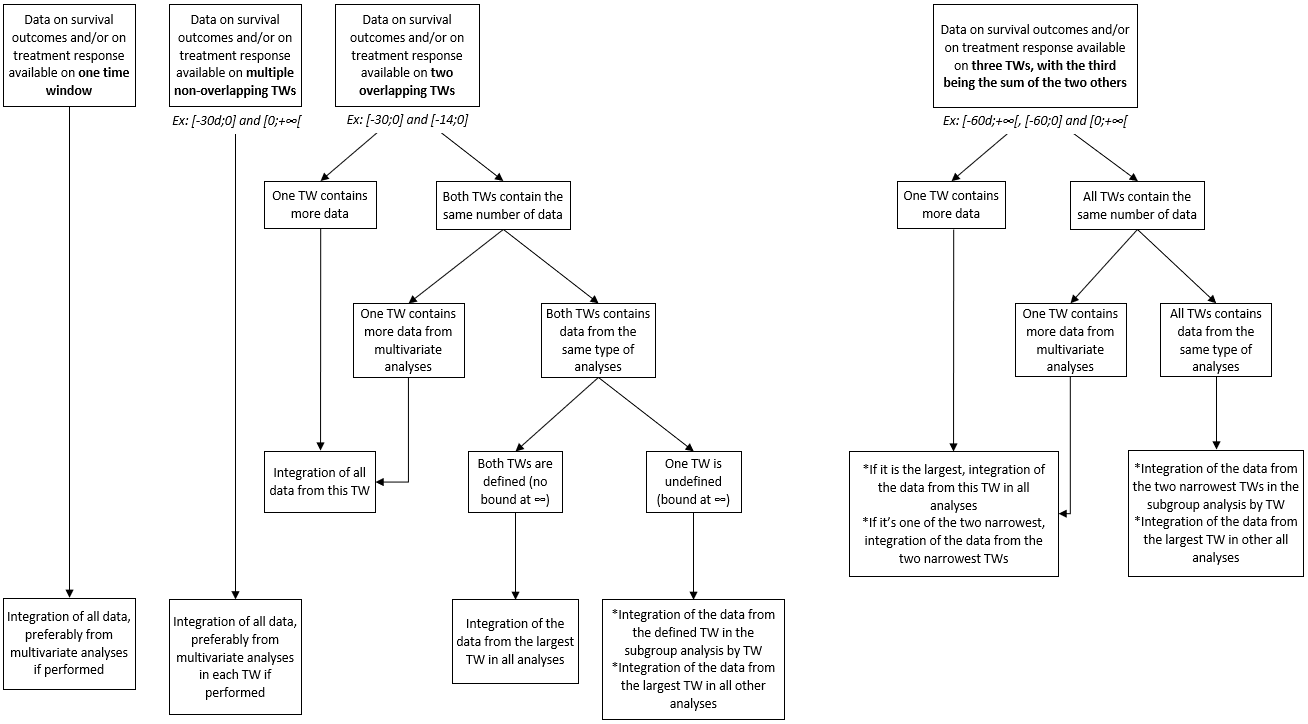


Supplementary Figure 1: Decision-making process for articles having reported data on multiple antibiotic exposure time windows.
TW: Time Window.

Supplementary Table 1: Evaluation of the included studies with the Newcastle-Ottawa Scale for cohort studies. Even if several studies were prospective, they were evaluated with the same grid in order to have a comparable evaluation. Of note, the evaluation is based on the published information only and may not reflect the true quality of the study itself.

|  | **First Author** | **Year of Publication** | **Format** | **Newcastle Ottawa Scale** | | |  |
| --- | --- | --- | --- | --- | --- | --- | --- |
|  |  |  |  | Selection | Comparability | Outcomes | Total score |
| **Non-Small Cell Lung Cancer** | Ahmed Y. | 2020 | Poster | **00 | 00 | **0 | 4 |
|  | Bagley^1^ | 2019 | Abstract | ***0 | ** | *00 | 6 |
|  | Castello | 2021 | Paper | ***0 | ** | **0 | 7 |
|  | Castro-Balado | 2021 | Abstract | **00 | 0* | 0*0 | 4 |
|  | Conde-Estévez | 2021 | Paper | ***0 | ** | **0 | 7 |
|  | Cortellini - Pembrolizumab monotherapy | 2021 | Paper | ***0 | ** | **0 | 7 |
|  | Cortellini - Chemo-immunotherapy | 2021 | Paper | ***0 | ** | **0 | 7 |
|  | Derosa | 2018 | Paper | ***0 | ** | *** | 8 |
|  | Derosa | 2022 | Paper | ***0 | ** | **0 | 7 |
|  | Fang | 2022 | Paper | ***0 | 00 | **0 | 5 |
|  | Forde | 2020 | Abstract | ***0 | 00 | *00 | 4 |
|  | Galli | 2019 | Paper | ***0 | ** | **0 | 7 |
|  | Geum | 2021 | Paper | ***0 | ** | **0 | 7 |
|  | Hakozaki | 2019 | Paper | ***0 | ** | **0 | 7 |
|  | Hamada | 2021 | Paper | **00 | ** | **0 | 6 |
|  | Hogue | 2019 | Abstract | ***0 | 00 | *00 | 4 |
|  | Hossain | 2020 | Abstract | ***0 | 00 | *00 | 4 |
|  | Hopkins | 2022 | Paper | ***0 | ** | **0 | 7 |
|  | Huemer | 2019 | Paper | ***0 | ** | *** | 8 |
|  | von Itzstein | 2022 | Paper | ***0 | *0 | **0 | 6 |
|  | Jin | 2019 | Paper | ***0 | 0* | *** | 7 |
|  | Kim H. | 2019 | Paper | ***0 | ** | **0 | 7 |
|  | Kostine^1^ | 2021 | Paper | ***0 | 00 | **0 | 5 |
|  | Kulkarni^1^ | 2020 | Paper | ***0 | ** | *00 | 6 |
|  | Lu | 2020 | Paper | ***0 | ** | **0 | 8 |
|  | Medjebar | 2020 | Paper | ***0 | ** | **0 | 7 |
|  | Metges | 2018 | Poster | ***0 | 00 | **0 | 5 |
|  | Mielgo Rubio | 2018 | Poster | ***0 | ** | **0 | 7 |
|  | Mielgo Rubio | 2019 | Abstract | ***0 | 0* | **0 | 6 |
|  | Nyein | 2022 | Paper | ***0 | ** | **0 | 7 |
|  | Ochi | 2021 | Paper | ***0 | ** | **0 | 7 |
|  | Ouaknine Krief | 2019 | Paper | ***0 | ** | **0 | 7 |
|  | Peng | 2021 | Paper | **** | ** | **0 | 8 |
|  | Pinato^1^ | 2019 | Paper | ***0 | ** | **0 | 7 |
|  | Qiu | 2022 | Paper | ***0 | 00 | **0 | 5 |
|  | Ren^1^ | 2021 | Poster | ***0 | 00 | **0 | 5 |
|  | Riudavets | 2019 | Poster | **00 | 00 | *00 | 3 |
|  | Rounis | 2021 | Paper | ***0 | ** | *00 | 6 |
|  | Ruiz-Patiño | 2020 | Paper | ***0 | 00 | **0 | 5 |
|  | Schett | 2019 | Paper | ***0 | ** | *** | 8 |
|  | Spakowicz^1^ | 2020 | Paper | ***0 | ** | **0 | 7 |
|  | Stokes | 2021 | Poster | **** | 00 | **0 | 6 |
|  | Sun | 2022 | Paper | ***0 | 00 | *** | 6 |
|  | Svaton | 2020 | Paper | ***0 | ** | **0 | 7 |
|  | Thompson | 2017 | Abstract | **00 | 0* | *00 | 3 |
|  | Do | 2018 | Abstract | **00 | 00 | *00 | 3 |
|  | Tomita | 2020 | Paper | ***0 | ** | *00 | 6 |
|  | Verschueren | 2021 | Paper | ***0 | ** | **0 | 7 |
|  | Zhang | 2021 | Paper | **00 | ** | **0 | 6 |
|  | Zhao | 2019 | Paper | ***0 | ** | **0 | 7 |
| **Urothelial Carcinoma** | Agarwal | 2019 | Poster | ***0 | ** | **0 | 7 |
|  | Braun | 2022 | Poster | ***0 | 00 | **0 | 5 |
|  | Fukuokaya | 2022 | Paper | ***0 | ** | **0 | 7 |
|  | Hoffman-Censits | 2020 | Poster | ***0 | 0* | **0 | 6 |
|  | Hopkins | 2022 | Paper | ***0 | ** | *** | 8 |
|  | Iida | 2022 | Abstract | **00 | 00 | *00 | 3 |
|  | Ishiyama | 2021 | Paper | ***0 | ** | *00 | 6 |
|  | Khan M. | 2020 | Poster | **00 | 00 | **0 | 4 |
|  | Okuyama | 2022 | Paper | **00 | ** | **0 | 6 |
|  | Routy | 2018 | Paper | **00 | ** | **0 | 6 |
|  | Ruiz-Bañobre | 2021 | Paper | ***0 | ** | *00 | 6 |
|  | Tomisaki | 2022 | Abstract | ***0 | ** | *0* | 7 |
|  | Weinstock | 2020 | Poster | ***0 | 0* | **0 | 6 |
| **Melanoma** | Elkrief | 2019 | Paper | ***0 | ** | **0 | 7 |
|  | Hemadri | 2019 | Poster | ***0 | 00 | **0 | 5 |
|  | Kapoor V. | 2019 | Abstract | **00 | 0* | *00 | 4 |
|  | Mohiuddin | 2020 | Paper | ***0 | ** | *** | 8 |
|  | Poizeau | 2022 | Paper | ***0 | 0* | *** | 7 |
|  | Swami | 2020 | Paper | ***0 | *0 | *** | 7 |
| **Renal Cell Carcinoma** | Braun | 2022 | Poster | ***0 | 00 | **0 | 5 |
|  | Derosa | 2021 | Poster | ***0 | ** | **0 | 7 |
|  | Ernst | 2021 | Poster | ***0 | 00 | **0 | 5 |
|  | Guven | 2021 | Paper | ***0 | 0* | *0* | 6 |
|  | Lalani | 2019 | Paper | ***0 | ** | *** | 8 |
|  | Ueda | 2019 | Paper | **00 | ** | **0 | 6 |
| **Hepatocellular Carcinoma** | Alshammari | 2021 | Abstract | **00 | 00 | *00 | 3 |
|  | Fessas | 2021 | Paper | **** | ** | **0 | 8 |
|  | Jun | 2020 | Poster | ***0 | 00 | *00 | 4 |
|  | Pinato* | 2022 | Poster | ***0 | 0* | **0 | 6 |
|  | Shen | 2021 | Paper | ***0 | 00 | *00 | 4 |
|  | Spahn | 2020 | Paper | **** | 00 | **0 | 6 |
| **Esophagogastric/ Gastric Cancer** | Greally | 2019 | Paper | ***0 | ** | *0* | 7 |
|  | Guo | 2020 | Paper | ***0 | ** | **0 | 7 |
|  | Jung* | 2021 | Poster | **00 | *0 | **0 | 5 |
|  | Kim J. H. | 2021 | Paper | ***0 | ** | **0 | 7 |
| **Head & Neck Cancer** | Plana | 2020 | Poster | ***0 | 0* | **0 | 6 |
|  | Vellanki | 2020 | Abstract | ***0 | ** | *00 | 6 |
| **Colorectal Cancer** | Serpas Higbie | 2022 | Paper | ***0 | ** | *** | 8 |
| **Cutaneous Squamous Cell Carcinoma** | Baggi | 2021 | Paper | ***0 | ** | *00 | 6 |
| **Gynecologic Cancers** | Chambers | 2021 | Paper | ***0 | ** | *00 | 6 |
| **Hodgkin Lymphoma** | Hwang | 2020 | Paper | **** | 0* | **0 | 7 |
| **Aggregated** | Abu-Sbeih | 2019 | Paper | ***0 | 0* | **0 | 6 |
|  | Ahmed J. | 2018 | Paper | ***0 | 0* | *00 | 5 |
|  | Araujo | 2021 | Poster | ***0 | ** | **0 | 7 |
|  | Eng | 2021 | Poster | **00 | 0* | **0 | 5 |
|  | Gaucher | 2021 | Paper | **** | ** | **0 | 8 |
|  | Giordan | 2021 | Paper | ***0 | ** | *00 | 6 |
|  | Iglesias‑Santamaría | 2019 | Paper | **** | 0* | **0 | 7 |
|  | Kapoor A. | 2020 | Paper | ***0 | ** | **0 | 7 |
|  | Khan U. | 2021 | Paper | ***0 | ** | *00 | 6 |
|  | Masini | 2019 | Abstract | ***0 | *0 | *00 | 6 |
|  | Ng Wei Qi | 2021 | Poster | ***0 | 00 | **0 | 5 |
|  | Pérez-Ruiz | 2020 | Paper | ***0 | ** | *00 | 6 |
|  | Sen | 2018 | Abstract | **00 | 00 | *00 | 3 |
|  | Tinsley | 2019 | Paper | **** | ** | *00 | 7 |
|  | Vick | 2020 | Poster | ***0 | 00 | *00 | 4 |
|  | Vitorino | 2021 | Poster | **00 | 00 | *00 | 3 |

^1^This article provided multiple cohorts.

**Supplementary Table 2: Characteristics of studies and patients included in the meta-analysis.** ABX, Antibiotic; CRC, Colorectal Cancer; CSCC, Cutaneous Squamous Cell Carcinoma; GC, Gynecologic Cancers, HCC, Hepatocellular Carcinoma; HL, Hodgkin Lymphoma; H&N Cancer, Head & Neck Cancer; ICI; Immune Checkpoint Inhibitor; MM, Melanoma; MV, Multivariate; NSCLC, Non-Small Cell Lung Cancer; ORR, Objective Response Rate, OS, Overall Survival; PFS, Progression-Free Survival; PD, Progressive Disease Rate; RCC, Renal Cell Cancer; RWE, Real-World Evidence; TW, Time Window; UC, Urothelial Carcinoma; UK, United Kingdom; USA, United States of America; UV, Univariate.

| **Cancer Type** | **First Author** | **Year and Type of Publi-cation** | **Study Design, Data Source** | **Study Country** | **Study Period** | **Number of Patients** | **Cancer Stage** | **Immunotherapy Treatment Characteristics** | | | **Antibiotherapy Characteristics** | | **Outcomes & Type of Analyses** |
| --- | --- | --- | --- | --- | --- | --- | --- | --- | --- | --- | --- | --- | --- |
|  |  |  |  |  |  |  |  | **ICI class** | **Treatment Scheme** | **Line of treatment** | **Number of ABX Users** | **ABX exposure TW (days)** |  |
| **NSCLC** | Ahmed Y. | 2020, poster | Retrospective analysis of medical records (RWE) | Ireland | 06/2016 - 05/2019 | 141 | Advanced | Anti-PD-1 | Monotherapy | Multiple | 53 (37.5%) | [-28;28] | ↓ OS (MV) &  ↓ PFS (UV) |
|  | Bagley | 2019, abstract | Retrospective analysis of FlatIron Electronic Health Record database (RWE) | USA | / | 1960 | Advanced | Monotherapy or in combination with chemotherapy | Monotherapy or in combination with chemotherapy | 1^st^ line | 61 (3.1%) | [-42;28] | ↓ OS (MV) |
|  | Castello | 2021, paper | Retrospective analysis of medical records from patients who were prospectively enrolled in clinical trials | Italy | 12/2015 - 05/2019 | 50 | Advanced | Mainly Anti-PD-1 | Mainly Monotherapy | Multiple | 20 (40%) | [-30;30] | ↓ OS (UV),  ↓ PFS (MV),  ↓ ORR &  ↑ PD (UV) |
|  | Castro-Balado | 2021, abstract | Retrospective analysis of medical records (RWE) | Spain | 07/2017 - 01/2020 | 49 | Metastatic | Anti-PD-1 | Monotherapy | 1^st^ line | 17 (34.7%) | [-30;30] | ↓ OS & ↓ PFS (MV) |
|  | Conde-Estévez | 2021, paper | Retrospective analysis of medical records (RWE) | Spain | 12/2015 - 05/2018 | 70 | Advanced or Recurrent | Mainly Anti-PD-1 | Monotherapy | Mostly 2^nd^ line | 39 (55.7%) | [-90;0] | ↓ OS & ↓ PFS (MV) |
|  | Cortellini - Pembrolizumab monotherapy | 2021, paper | Retrospective analysis of medical records (RWE) | Interna-tional (mainly Italy, but also Switzer-land, and The Nether-lands) | 01/2013 - 05/2020 | 950 | Metastatic | Anti-PD-1 | Monotherapy | 1^st^ line | 131 (13.8%) | [-30;0] | ↓ OS & ↓ PFS (MV), ↓ ORR (MV) |
|  | Cortellini - Chemo-immunotherapy | 2021, paper | Retrospective analysis of medical records (RWE) | Interna-tional (UK, Spain, France, Belgium, Italy, US) | 12/2014 - 10/2020 | 302 | Stage IV | Anti-PD-1 | Combination with chemotherapy | 1^st^ line | 47 (15.6%) 117 (38.7%) | [-30;0] [0;∞[ | ↓ OS & ↓ PFS (MV), ↓ ORR (MV) ↓ OS & ↓ PFS (MV) |
|  | Derosa | 2018, paper | Retrospective analysis of medical records (RWE & Clinical trials for 23% of the patients) | USA | Until 03/2017 | 239 | Advanced | Multiple | Mainly Monotherapy | Multiple | 48 (20%) | [-30;0] | ↓ OS & ↓ PFS (MV), ↓ ORR & ↑ PD (UV) |
|  | Derosa | 2022, paper | Prospective observational clinical trial | France, Canada | 12/2015 - 11/2019 | 338 | Advanced | Multiple | Mainly Monotherapy | Multiple | 69 (20%) | [-60;0] | ↓ OS (MV) |
|  | Do | 2018, abstract | Retrospective analysis of medical records (RWE) | USA | 2015 - 2017 | 109 | Advanced lung cancer | Anti-PD-1 | Monotherapy | / | 87 (79.8%) | [-30;∞[ | ↓ OS (UV) |
|  | Fang | 2022, paper | Retrospective analysis of medical records from patients who were prospectively enrolled in clinical trials | China | 12/2015 - 08/2017 | 85 | Advanced or Recurrent | Anti-PD-1 | Monotherapy | ≥ 2^nd^ line | 17 (20%) | [0;90] | ↑ PFS (UV), ↓ ORR & ↓ PD (UV) |
|  | Forde | 2020, abstract | Retrospective analysis of medical records (RWE) | Ireland | / | 86 | Advanced | Mainly Anti-PD-1 | Monotherapy | Multiple | 34 (40%) | [-30;60] | ↓ OS & ↓ PFS (UV) |
|  | Galli | 2019, paper | Retrospective analysis of medical records (RWE) | Italy | 04/2013 - 01/2018 | 157 | Metastatic | Multiple | Mainly Monotherapy | Mainly ≥ 2^nd^ line | 27 (17.2%) | [-30;90] | ↓ OS & ↓ PFS (UV), ↓ ORR & ↑ PD (UV) |
|  | Geum | 2021, paper | Retrospective analysis of medical records (RWE) | South Korea | 07/2015 - 06/2018 | 140 | Mainly Stage IV | Anti-PD-1 | Monotherapy | Multiple | 70 (50%) | [-30;∞[ | ↓ OS & ↑ PFS (UV) |
|  | Hakozaki | 2019, paper | Retrospective analysis of medical records from patients who were prospectively enrolled in clinical trials | Japan | 01/2016 - 04/2017 | 90 | Stage IV or Recurrent | Anti-PD-1 | Monotherapy | ≥ 2^nd^ line | 13 (14.4%) | [-30;0] | ↓ OS (MV), ↓ PFS (UV) |
|  | Hamada | 2021, paper | Retrospective analysis of medical records (RWE) | Japan | 01/2016 - 12/2019 | 69 | Mainly Stage IV | Anti-PD-1 | Monotherapy | Multiple | 18 (26.1%) | [-21;21] | ↓ OS & ↓ PFS (MV), ↓ ORR & ↑ PD (UV) |
|  | Hogue | 2019, abstract | Retrospective analysis of medical records (RWE) | USA | 2015 - 2019 | 161 | Stage IV or Relapsed | Anti-PD-1 | Monotherapy | ≥ 2^nd^ line | 58 (36%) 33 (20%) | [-90;0] [0;∞[ | ↓ OS & ↓ PFS (MV) ↑ OS & ↑ PFS (MV) |
|  | Hopkins | 2022, paper | Retrospective analysis of medical records from patients who were prospectively enrolled in clinical trials | Interna-tional | 2013-2018 | 2723 | Advanced | Anti-PD-L1 | Monotherapy or in combination with chemotherapy | Multiple | 194 (7.1%) 518 (19%) | [-30;0] [0;30] | ↓ OS & ↑ PFS (MV) ↓ OS & ↓ PFS (MV) |
|  | Hossain | 2020, abstract | Retrospective analysis of medical records (RWE) | Australia | 2015 - 2019 | 63 | Advanced | ICI | / | / | 18 (28.6%) | [-14;42] | ↓ OS & ↓ PFS (UV) |
|  | Huemer | 2019, paper | Retrospective analysis of medical records (RWE & Clinical trials) | Austria | 05/2015 - 01/2018 | 142 | Mainly Stage IV | Mainly Anti-PD-1 | Mainly monotherapy | Multiple | 62 (44%) | [-30;30] | ↑ OS & ↓ PFS (UV) |
|  | von Itzstein | 2022, paper | Retrospective analysis of medical records from patients who were prospectively enrolled in clinical trials | USA | / | 133 | Mainly Stage IV | ICI | / | / | 19 (14.3%) 35 (26.3%) | [-42;0] [0;42] | ↓ OS & ↓ PFS (MV) ↓ OS & ↓ PFS (MV) ↓ ORR & ↑ PD (UV) on [-42;42] |
|  | Jin | 2019, paper | Retrospective analysis of medical records from patients who were prospectively enrolled in clinical trials | China | / | 37 | Stage IIIb or Stage IV | Anti-PD-1 | Monotherapy | ≥ 2^nd^ line | 11 (29.7%) | [-30;30] | ↑ PFS (UV), ↑ PD (UV) |
|  | Kim H. | 2019, paper | Retrospective analysis of medical records (RWE) | South Korea | 02/2012 - 05/2018 | 131 | Mainly Stage IV | Mainly Anti-PD-1 | Mainly monotherapy | Multiple | 60 (45.8%) | [-60;0] | ↓ OS & ↓ PFS (MV), ↓ ORR & ↑ PD (UV) |
|  | Kostine | 2021, paper | Retrospective analysis of medical records (RWE) | France | 05/2015 - 09/2017 | 149 | Advanced | Mainly Anti-PD-1 | / | / | 41 (27%) | [-30;30] | ↓ OS & ↓ PFS (UV) |
|  | Kulkarni | 2020, paper | Retrospective analysis of medical records (RWE) | USA | 05/2015 - 12/2017 | 140 | Metastatic | Anti-PD-1 | Monotherapy | Mainly ≥ 2^nd^ line | 54 (39%) | [-30;42] | ↓ OS & ↓ PFS (UV), ↑ PD (UV) |
|  | Lu | 2020, paper | Retrospective analysis of medical records (RWE) | Taiwan | 01/2016 - 03/2019 | 340 | / | ICI | Mainly Monotherapy | Multiple | 128 (38%) | [-30;0] | ↓ OS (MV) |
|  | Medjebar | 2020, paper | Retrospective analysis of medical records (RWE) | France | 06/2013 - 12/2018 | 178 | Metastatic | Mainly Anti-PD-1 | Monotherapy | Mainly ≥ 2^nd^ line | 31 (17.8%) | [0;∞[ | ↑ OS & ↑ PFS (MV) |
|  | Metges | 2018, poster | Retrospective analysis of the French Regional Health Insurance System & of the French Social Security System (RWE) | France | 01/2016 - 12/2017 | 325 | Lung cancer (no precision) | Anti-PD-1 | Monotherapy | / | 153 (47.1%) | [-60;∞[ | ↑ OS (UV) |
|  | Mielgo Rubio | 2018, poster | Retrospective analysis of medical records (RWE) | Spain | 09/2016 - 03/2019 | 168 | Stage III-IV | Anti-PD-1 | Monotherapy | ≥ 2^nd^ line | 79 (47%) | [-60;30] | ↓ OS (MV), ↓ PFS (UV) |
|  | Mielgo Rubio | 2019, poster | Retrospective analysis of medical records (RWE) | Spain | 09/2016 - 03/2019 | 121 | Metastatic | Anti-PD-1 | Monotherapy | 1^st^ line | 55 (45%) | [-60;30] | ↓ OS & ↓ PFS (MV), ↑ PD (UV) |
|  | Nyein | 2022, paper | Retrospective analysis of medical records (RWE) | USA | 01/2011 - 03/2017 | 256 | Stage III or Stage IV | ICI | Mixed | / | 46 (18%) | [-60;30] | ↓ OS (MV), ↓ ORR (UV) |
|  | Ochi | 2021, paper | Retrospective analysis of medical records (RWE) | Japan | 12/2015 - 05/2018 | 531 | Mainly Stage IV | Anti-PD(L)1 | Monotherapy | Multiple | 98 (19%) | [-60;30] | ↓ OS & ↓ PFS (UV) |
|  | Ouaknine Krief | 2019, paper | Retrospective analysis of medical records (RWE) | France | 07/2014 - 09/2017 | 72 | Mainly Stage IV | Anti-PD-1 | Monotherapy | ≥ 2^nd^ line | 30 (42%) | [-60;30] | ↓ OS & ↓ PFS (MV), ↑ ORR (UV) |
|  | Peng | 2021, paper | Retrospective analysis of medical records (RWE) | USA | 09/2014 - 08/2019 | 117 | Stage IV | Anti-PD-1 | Mainly Monotherapy | Multiple | 41 (35%) | [-30;30] | ↓ OS & ↓ PFS (UV) |
|  | Pinato | 2019, paper | Retrospective analysis of medical records entered into a prospectively maintained database (RWE) | UK | 01/2015 - 01/2018 | 119 | Mainly metastatic | ICI | / | Multiple | 29 (24.4%) | [-30;0] | ↓ OS (UV) |
|  | Qiu | 2022, paper | Retrospective analysis of medical records (RWE) | China | 10/2018 - 06/2021 | 148 | Advanced | Mainly Anti-PD-1 | Mixed | Multiple | 80 (54%) | [-60;60] | ↓ OS & ↓ PFS (UV) |
|  | Ren | 2021, poster | Retrospective analysis of medical records from patients who were prospectively enrolled in clinical trials | Interna-tional | 2015 - 2020 | 98 | Advanced | Anti-PD-1 | Monotherapy | Multiple | 27 (27.6%) | [-30;30] | ↓ OS (UV) |
|  | Riudavets | 2019, poster | Retrospective analysis of medical records (RWE) | Spain | 03/2013 - 08/2018 | 267 | Advanced | ICI | Mainly Monotherapy | Multiple | 141 (52.8%) | [-90;∞[ | ↑ OS (UV) |
|  | Rounis | 2021, paper | Prospective observational clinical trial | Greece | 11/2017 - 11/2019 | 66 | Metastatic | Anti-PD(L)1 | Monotherapy | 2^nd^ line | 34 (51.5%) | [-30;84] | ↓ OS & ↓ PFS (UV), ↓ ORR (UV) |
|  | Ruiz-Patiño | 2020, paper | Retrospective analysis of medical records (RWE) | Mexico, Colombia Costa Rica, Peru | 06/2013 - 01/2018 | 140 | Metastatic or Inoperable | Mainly Anti-PD-1 | Mainly Monotherapy | Multiple | 32 (22.9%) | [-30;∞[ | ↓ OS (MV), ↓ PFS (UV), ↓ ORR (UV) |
|  | Schett | 2019, paper | Retrospective analysis of medical records (RWE) | Switzer-land | 01/2013 - 12/2017 | 218 | Mainly Stage IV | Mainly Anti-PD-1 | Mainly Monotherapy | Multiple | 33 (15.1%) N/A | [-60;0] [0;∞[ | ↓ OS (MV), ↓ PFS (UV), ↓ ORR & ↑ PD (UV) ↓ OS & ↑ PFS (UV) |
|  | Spakowicz | 2020, paper | Retrospective analysis of medical records (RWE) | USA | 2011 - 2017 | 196 | Mainly Stage IV | ICI | / | / | 70 (35.7%) | [-28;28] | ↓ OS (UV) |
|  | Stokes | 2021, poster | Retrospective analysis of medical records (RWE) of Veterans | USA | 2010 - 2018 | 3634 | / | Mainly Anti-PD-1 | / | / | 762 (21.0%) 970 (26.7%) | [-30;0] [0;60[ | ↓ OS (MV) ↓ OS (MV) |
|  | Sun | 2022, paper | Retrospective analysis of medical records (RWE) | China | 01/2015 - 03/2021 | 245 | Mainly Stage IV | Mainly Anti-PD-1 | Monotherapy or in combination with chemotherapy | Multiple | 72 (29.4%) | [-30;30] | ↓ ORR & ↑ PD (UV) |
|  | Svaton | 2020, paper | Retrospective analysis of medical records (RWE) | Czech Republic | 2015 - 2019 | 224 | Stage III or Stage IV | Anti-PD-1 | Monotherapy | Multiple | 27 (12.1%) | [-30;30] | ↑ OS & ↑ PFS (MV) |
|  | Thompson | 2017, abstract | Retrospective analysis of medical records (RWE) | USA | / | 74 | Metastatic | Anti-PD-1 | / | / | 18 (24%) | [-42;0] | ↓ OS & ↓ PFS (MV), ↑ ORR (UV) |
|  | Tomita | 2020, paper | Retrospective analysis of medical records (RWE) | Japan | 01/2016 - 05/2019 | 79 | Advanced | Mainly Anti-PD-1 | Mainly Monotherapy | Multiple | 24 (30%) | [-60;0] | ↓ OS & ↓ PFS (UV) |
|  | Verschueren | 2021, paper | Retrospective analysis of medical records (RWE) - Matched cohort study | The Nether-lands | 01/2015 - 01/2019 | 221 | Stage IV | Mainly Anti-PD-1 | Monotherapy | Multiple | 35 (15.8%) | [-30;30] | ↓ OS (MV) |
|  | Zhang | 2021, paper | Prospective observational clinical trial | Spain | 11/2017 - 06/2019 | 69 | Stage III or Stage IV | ICI | Monotherapy | Multiple | 16 (23.2%) | [-90;0] | ↑ OS & ↓ PFS (UV), ↑ PD (UV) |
|  | Zhao | 2019, paper | Retrospective analysis of medical records (RWE & Clinical trials for 69.7% of the patients) | China | 01/2016 - 01/2018 | 109 | Advanced | Mainly Anti-PD-1 | Mixed | Multiple | 20 (18.3%) | [-30;30] | ↓ OS & ↓ PFS (MV), ↓ ORR & ↑ PD (UV) |
| **UC** | Agarwal | 2019, poster | Retrospective analysis of medical records (RWE) | USA | / | 101 | Metastatic | Anti-PD(L)1 | / | Multiple | 26 (25.7%) | [-30;∞[ | ↓ OS (MV) |
|  | Braun | 2022, poster | Retrospective analysis of FlatIron Electronic Health Record database (RWE) | USA | / | 1483 | Advanced | / | / | 1^st^ line | 182 (12.3%) | [0;∞[ | ↓ OS (MV) |
|  | Fukuokaya | 2022, paper | Retrospective analysis of medical records (RWE) | Japan | 04/2018 - 04/2021 | 227 | Metastatic | Anti-PD-1 | Monotherapy | ≥ 2^nd^ line | 61 (26.9%) | [-30;30] | ↓ OS & ↓ PFS (MV), ↓ ORR (MV) |
|  | Hoffman-Censits | 2020, poster, | Retrospective analysis of Truven database (RWE) | USA | 01/2016 - 12/2018 | 350 | Metastatic | Mainly Anti-PD-1 | / | 1^st^ line | 167 (47.8%) 169 (48.3%) | [-90;0] [0;∞[ | ↓ OS (UV), ↓ PFS (MV) ↓ PFS (MV) |
|  | Hopkins | 2022, paper | Retrospective analysis of medical records from patients who were prospectively enrolled in clinical trials | Interna-tional | 2014 - 2017 | 896 | Locally Advanced & Metastatic | Anti-PD-L1 | Monotherapy | ≥ 2^nd^ line | 235 (26.2%) | [-30;30] | ↓ OS & ↓ PFS (UV) |
|  | Iida | 2022, abstract | Retrospective analysis of medical records (RWE) | Japan | 01/2018 - 01/2021 | 115 | Metastatic | Anti-PD-1 | Monotherapy | 2^nd^ line | N/A | [-30;∞[ | ↓ PFS (MV) |
|  | Ishiyama | 2021, paper | Retrospective analysis of medical records (RWE) | Japan | 01/2018 - 10/2020 | 67 | Metastatic or Relapsed | Anti-PD-1 | Monotherapy | ≥ 2^nd^ line | 15 (22%) | [-60;30] | ↓ OS & ↓ PFS (MV), ↓ ORR & PD (UV) |
|  | Khan M. | 2020, poster | Retrospective analysis of medical records (RWE) | USA | 2015 - 2020 | 130 | Metastatic | Anti-PD(L)1 | / | / | 70 (53.8%) 44 (33.8%) | [-60;60] [0;60[ | ↓ OS (UV), ↑ PFS (U) ↓ OS (UV), ↓ PFS (U) |
|  | Okuyama | 2022, paper | Retrospective analysis of medical records (RWE) | Japan | 08/2015 - 04/2021 | 155 | Locally Advanced & Metastatic | Mainly Anti-PD-1 | / | ≥ 2^nd^ line | 71 (45.8%) | [-30;∞[ | ↓ OS & ↑ PFS (MV), ↓ ORR (UV) |
|  | *Ren* | 2021, poster | Retrospective analysis of medical records from patients who were prospectively enrolled in clinical trials | Interna-tional | 2015 - 2020 | 143 | Locally Advanced & Metastatic | Anti-PD-1 | Monotherapy | / | 38 (26.6%) | [-30;30] | ↓ OS (UV) |
|  | Routy | 2018, paper | Retrospective analysis of medical records from patients who were prospectively enrolled in clinical trials | France | 2012 - 2020 | 42 | / | Anti-PD(L)1 | / | ≥ 2^nd^ line | 12 (28.6%) | [-60;30] | ↓ OS (UV), ↓ PFS (MV) |
|  | Ruiz-Bañobre | 2021, paper | Retrospective analysis of medical records (RWE) | Spain | 06/2016 - 02/2020 | 119 | Metastatic | Mainly Anti-PD-L1 | Monotherapy | Multiple | 11 (9%) | [-30;0] | ↓ OS & ↓ PFS (MV), ↓ ORR (UV) |
|  | *Spakowicz* | 2020, paper | Retrospective analysis of medical records (RWE) | USA | 2011 - 2017 | 38 | Mainly Stage IV | ICI | / | / | 20 (52.6%) | [-28;28] | ↓ OS (UV) |
|  | Tomisaki | 2022, paper | Retrospective analysis of medical records (RWE) | Japan | 03/2018 - 03/2021 | 40 | Advanced | Anti-PD-1 | Monotherapy | ≥ 2^nd^ line | 12 (30%) | [-30;30] | ↑ OS & ↑ PFS (MV) |
|  | Weinstock | 2020, poster | Retrospective analysis of medical records from patients who were prospectively enrolled in clinical trials | / | / | 1747 | Advanced | ICI | / | Multiple | 482 (27.6%) | [-30;30] | ↑ OS & ↑ PFS (UV) |
| **MM** | *Bagley* | 2019, abstract | Retrospective analysis of FlatIron Electronic Health Record database (RWE) | USA | / | 1177 | Advanced | ICI | Monotherapy or in combination with chemotherapy | 1^st^ line | 23 (2%) | [-42;28] | ↑ OS (MV) |
|  | Elkrief | 2019, paper | Retrospective analysis of medical records (RWE & Clinical trials for 20.3% of the patients) | Canada | Unknown - 05/2018 | 74 | Stage IV | Mainly Anti-PD-1 | Mixed | Mainly 1^st^ line | 10 (13.5%) | [-30;0] | ↓ OS & ↓ PFS (MV), ↓ ORR & ↑ PD (UV) |
|  | Hemadri | 2019, poster | Retrospective analysis of medical records (RWE) | USA | 2014 - 2018 | 172 | Stage IV | Anti-PD-1 | / | Multiple | 29 (17%) | [0;∞[ | ↓ OS & ↓ PFS (UV) |
|  | Kapoor V. | 2019, abstract | Retrospective analysis of medical records (RWE) | Mexico | 11/2010 - 10/2017 | 108 | Stage IV | Anti-PD-1 or Anti-CTLA-4 | Monotherapy or ICI Combination | / | 46 (42.6%) | [-180;30] | ↑ PD (UV) |
|  | *Kostine* | 2021, paper | Retrospective analysis of medical records (RWE) | France | 05/2015 - 09/2017 | 293 | Advanced | Mainly Anti-PD-1 | / | / | 42 (14.3%) | [-30;30] | ↓ OS & ↓ PFS (UV) |
|  | Mohiuddin | 2020, paper | Retrospective analysis of medical records (RWE) | USA | 2008 - 2019 | 568 | Stage III or Stage IV | Anti-PD-1 or Anti-CTLA-4 | Monotherapy or ICI Combination | Mainly ≥ 2^nd^ line | 114 (20.1%) | [-90;0] | ↓ OS (MV) |
|  | *Pinato* | 2019, paper | Retrospective analysis of medical records entered into a prospectively maintained database (RWE) | UK | 01/2015 - 01/2018 | 38 | Mainly metastatic | ICI | / | Multiple | 17 (44.7%) | [-30;0] | ↓ OS (UV) |
|  | Poizeau | 2022, paper | Retrospective analysis of data prospectively collected in the French National Health Insurance database (SDNS) (RWE) | France | 06/2015 - 12/2017 | 2605 | Metastatic | Anti-PD-1 | Monotherapy | 1^st^ line | 749 (28.8%) | [-90;0] | ↓ OS (MV) |
|  | *Spakowicz* | 2020, paper | Retrospective analysis of medical records (RWE) | USA | 2011 - 2017 | 321 | Mainly Stage IV | ICI | / | / | 74 (25.2%) | [-28;28] | ↓ OS (UV) |
|  | Swami | 2020, paper | Retrospective analysis of medical records (RWE) | USA | 08/2012 - 07/2017 | 166 | Unresectable, Advanced or Metastatic | Anti-PD-1 | Mixed | Multiple | 30 (18.1%) | [-60;0] | ↓ OS & ↓ PFS (UV) |
| **RCC** | Braun | 2022, poster | Retrospective analysis of FlatIron Electronic Health Record database (RWE) | USA | / | 1805 | Advanced | ICI | / | 1^st^ line | 199 (11%) | [0;∞[ | ↓ OS & ↓ PFS (MV) |
|  | Derosa | 2021, poster | Prospective clinical trial | France | 02/2016 - 06/2017 | 707 | Metastatic | Anti-PD-1 | Monotherapy | ≥ 2^nd^ line | 104 (14.7%) | [-60;42] | ↓ OS (MV), ↓ PFS (UV), ↓ ORR & ↑ PD (UV) |
|  | Ernst | 2021, poster | Retrospective analysis of the International mRCC Database Consortium (IMDC) | USA | 2009 - 2020 | 427 | Metastatic | ICI | Monotherapy or Combination with Targeted Therapy | Multiple | 56 (13%) | [-60;0] | ↓ OS (MV), ↓ ORR (UV) |
|  | Can Guven | 2021, paper | Retrospective analysis of medical records (RWE) | Turkey | 04/2016 - 12/2019 | 93 | Metastatic | Anti-PD-1 | Monotherapy | ≥ 2^nd^ line | 31 (33.3%) | [-90;90] | ↓ OS & ↓ PFS (MV), ↓ ORR & PD ↑ (UV) |
|  | *Kostine* | 2021, paper | Retrospective analysis of medical records (RWE) | France | 05/2015 - 09/2017 | 83 | Advanced | Mainly Anti-PD-1 | / | / | 20 (24.1%) | [-30;30] | ↓ OS & ↓ PFS (UV) |
|  | *Kulkarni* | 2020, paper | Retrospective analysis of medical records (RWE) | USA | 05/2015 - 12/2017 | 55 | Metastatic | Anti-PD-1 | Monotherapy | Mainly ≥ 2^nd^ line | 24 (43.6%) | [-30;42] | ↓ OS & ↓ PFS (UV), ↑ PD (UV) |
|  | Lalani | 2019, paper | Retrospective analysis of medical records (RWE) | USA | 2009 - 2017 | 146 | Metastatic | Multiple | Monotherapy or ICI/Targeted Therapy Combination | Multiple | 31 (21%) | [-56;28] | ↓ OS & ↓ PFS (MV), ↓ ORR (UV) |
|  | *Spakowicz* | 2020, paper | Retrospective analysis of medical records (RWE) | USA | 2011 - 2017 | 104 | Mainly Stage IV | ICI | / | / | 34 (32.7%) | [-28;28] | ↓ OS (UV) |
|  | Ueda | 2019, paper | Retrospective analysis of medical records (RWE) | Japan | 11/2016 - 04/2019 | 31 | Metastatic | Mainly Anti-PD-1 | Mainly monotherapy | Mainly ≥ 2^nd^ line | 5 (16.1%) | [-30;0] | ↓ PFS (MV), ↓ ORR & ↑ PD (UV) |
| **HCC** | Alsham-mari | 2021, abstract | Retrospective analysis of medical records (RWE) | Saudi Arabia | / | 59 | Advanced | Anti-PD-1 | Monotherapy | ≥ 2^nd^ line | 20 (34%) | [-14;28] | ↓ ORR (UV) |
|  | Fessas | 2021, paper | Retrospective analysis of medical records from patients who were prospectively enrolled in clinical trials | Europe, North America, Asia | 2017 - 2019 | 402 | Advanced | ICI | Mainly monotherapy | Multiple | 155 (38.6%) | [-30;30] | ↓ OS & ↑ PFS (MV), ↑ ORR (MV), ↓ PD (UV) |
|  | Jun | 2020, poster | Retrospective analysis of medical records (RWE) | USA | / | 95 | Advanced | Anti-PD-1 | Monotherapy | / | 25 (26.3%) | [-60;0] | ↓ OS (MV) |
|  | Pinato - ICI mono-therapy | 2022, poster | Retrospective analysis of medical records from patients who were prospectively enrolled in clinical trials | Interna-tional | / | 258 | Un-resectable | ICI | Monotherapy | Multiple | 33 (12.8%) | [-30;30] | ↓ OS & ↑ PFS (MV) |
|  | *Pinato - Chemo-immuno-therapy* | 2022, poster | Retrospective analysis of medical records from patients who were prospectively enrolled in clinical trials | Interna-tional | / | 584 | Un-resectable | ICI | Combination with chemotherapy | Multiple | 102 (17.5%) | [-30;30] | ↓ OS & ↑ PFS (MV) |
|  | *Ren* | 2021, poster | Retrospective analysis of medical records from patients who were prospectively enrolled in clinical trials | Interna-tional | 2015 - 2020 | 317 | Advanced | Anti-PD-1 | Monotherapy | / | 36 (11.4%) | [-30;30] | ↓ OS (UV) |
|  | Shen | 2021, paper | Prospective study | Taiwan | 2017 - 2019 | 36 | Advanced | Anti-PD(L)1 | Mixed | Multiple | 4 (11.1%) | [-28;0] | ↓ OS (UV), ↓ ORR & ↑ PD (UV) |
|  | Spahn | 2020, paper | Retrospective analysis of medical records (RWE) | Germany Austria, Switzer-land | 08/2015 - 12/2019 | 99 | Advanced | Anti-PD-1 | Monotherapy | Multiple | 13 (13.1%) | [-30;30] | ↓ OS & ↑ PFS (UV), ↓ ORR & ↑ PD (UV) |
| (**Esophago)**  **Gastric Cancer** | Greally | 2019, paper | Retrospective analysis of medical records (RWE) | USA | 09/2013 - 05/2018 | 161 | Metastatic | ICI | Monotherapy or ICI Combination | Multiple | 14 (8.7%) | [-30;0] | ↓ OS & ↑ PFS (UV) |
|  | Guo | 2020, paper | Retrospective analysis of medical records (RWE) | Taiwan & China | 08/2015 - 12/2017 | 49 | Recurrent or Metastatic | Anti-PD(L)1 | Mixed | Mainly ≥ 2^nd^ line | 21 (42.9%) | [-60;30] | ↓ OS & ↑ PFS (MV) |
|  | Jung | 2021, poster | Prospective clinical trial | South Korea | 01/2014 - 06/2020 | 228 | Advanced | Anti-PD-1 | / | Multiple | 114 (50%) | [-28;0] | ↓ OS & ↑ PFS (MV), ↓ ORR & ↑ PD (UV) |
|  | Kim J. H. | 2021, paper | Retrospective analysis of medical records (RWE) | South Korea | 05/2016 - 12/2019 | 60 | Metastatic or Unresectable | Anti-PD-1 | Monotherapy | ≥ 2^nd^ line | 15 (25%) | [-30;0] | ↓ OS & ↑ PFS (MV), ↓ ORR & ↑ PD (UV) |
|  | *Ren* | 2021, poster | Retrospective analysis of medical records from patients who were prospectively enrolled in clinical trials | Interna-tional | 2015 - 2020 | 46 | Advanced | Anti-PD-1 | Monotherapy | / | 14 (30.4%) | [-30;30] | ↓ OS (UV) |
| **H&N Cancer** | Plana | 2020, poster | Retrospective analysis of medical records (RWE) | Spain | 2015 - 2019 | 74 | Recurrent or Metastatic | ICI | Mixed | Multiple | 23 (31.1%) | [-30;30] | ↓ OS (MV), ↑ PFS (UV), ↑ ORR & ↓ PD (UV) |
|  | *Spakowicz* | 2020, paper | Retrospective analysis of medical records (RWE) | USA | 2011 - 2017 | 64 | Mainly Stage IV | ICI | / | / | 19 (29.7%) | [-28;28] | ↓ OS (UV) |
|  | Vellanki | 2020, abstract | Retrospective analysis of medical records from patients who were prospectively enrolled in clinical trials | Interna-tional | / | 1037 | Recurrent or Metastatic | ICI | Monotherapy or ICI Combination | / | 372 (35.9%) | [-30;30] | ↓ OS & ↑ PFS (UV) |
| **CRC** | Serpas Higbie | 2022, paper | Retrospective analysis of medical records (RWE) | USA | 2013 - 2021 | 57 | Metastatic | Mainly Anti-PD-1 | Mainly monotherapy | Multiple | 19 (33.3%) | [-90;42] | ↓ ORR (UV) |
| **CSCC** | Baggi | 2021, paper | Retrospective analysis of medical records from patients treated within the named patient programme - compassionate use | Italy | 05/2019 - 02/2020 | 131 | Locally advanced or Metastatic | Anti-PD-1 | / | / | 8 (6.5%) | [-30;0] | ↑ PD (MV) |
| **GC** | Chambers | 2021, paper | Retrospective analysis of medical records (RWE) | USA | 01/2017 - 09/2020 | 101 | Multiple | Mainly Anti-PD-1 | Mainly monotherapy | Multiple | 23 (22.8%) 35 (34.7%) | [-30;0] [0;∞[ | ↓ OS & ↑ PFS (MV), ↓ ORR & ↑ PD (UV) ↑ OS & ↑ PFS (UV), ↓ ORR & ↓ PD (UV) |
| **HL** | Hwang | 2020, paper | Retrospective analysis of medical records (RWE) | USA | 01/2011 - 10/2018 | 62 | Multiple | Mainly Anti-PD-1 | Mainly monotherapy | Multiple | 20 (32%) 21 (34%) | [-90;0] [0;90] | ↑ PFS (MV) ↑ PFS (MV) |
| **Sarcoma** | *Spakowicz* | 2020, paper | Retrospective analysis of medical records (RWE) | USA | 2011 - 2017 | 45 | Mainly Stage IV | ICI | / | / | 14 (31.1%) | [-28;28] | ↓ OS (UV) |
| **Aggre-gated** | Abu-Sbeih | 2019, paper | Retrospective analysis of medical records (RWE) | USA | 01/2016 - 01/2018 | 826 | Mainly Stage IV | Multiple | Mainly monotherapy | / | 569 (69%) | [-90;∞[ | ↓ OS (UV) |
|  | Ahmed J. | 2018, paper | Retrospective analysis of medical records (RWE) | USA | 04/2014 - 12/2017 | 60 | Advanced | Mainly Anti-PD-1 | Mainly monotherapy | Multiple | 17 (28%) | [-14;14] | ↓ OS & ↑ PFS (UV), ↓ ORR (UV) |
|  | Araujo | 2021, poster | Retrospective analysis of medical records (RWE) | Brazil | / | 216 | Metastatic | Multiple | / | / | 34 (15.7%) 92 (42.6%) | [-60;0] [0;∞[ | ↓ OS & ↑ PFS (MV) ↓ OS & ↑ PFS (UV) |
|  | Eng | 2021, poster | Retrospective analysis of population-level administrative data for the province of Ontario (RWE) | Canada | 06/2012 - 10/2018 | 2737 | / | Mainly Anti-PD-1 | / | / | 520 (19%) | [-60;0] | ↓ OS (UV) |
|  | Gaucher | 2021, paper | Retrospective analysis of medical records (RWE) | France | 12/2010 - 12/2019 | 372 | Mainly metastatic | Anti-PD-1 or Anti-CTLA-4 | Mixed | Multiple | 100 (26.9%) | [-60;60] | ↓ OS (MV), ↑ ORR & ↓ PD (UV) |
|  | Giordan | 2021, paper | Retrospective analysis of medical records (RWE) | France | 01/2018 - 12/2019 | 138 | Mainly Stage IV | Anti-PD-1 | Mainly monotherapy | Multiple | 31 (22.5%) | [-60;0] | ↓ OS & ↑ PFS (MV), ↓ ORR (MV), ↑ PD (UV) |
|  | Iglesias‑Santamaría | 2019, paper | Retrospective analysis of medical records (RWE) | Spain | 06/2015 - 12/2018 | 102 | Mainly Stage IV | Mainly Anti-PD-1 | Mainly monotherapy | Multiple | 33 (32.3%) | [-28;28] | ↑ OS & ↑ PFS (UV), ↑ ORR & ↓ PD (UV) on [0;∞[ |
|  | Kapoor A. | 2020, paper | Retrospective analysis of medical records collected prospectively (RWE) | India | 08/2015 - 11/2018 | 155 | Advanced | Anti-PD-1 | Monotherapy | Multiple | 33 (21.2%) | [-14;60] | ↓ OS (UV) |
|  | *Kostine* | 2021, paper | Retrospective analysis of medical records (RWE) | France | 05/2015 - 09/2017 | 635 | Advanced | Mainly Anti-PD-1 | / | / | 150 (23.6%) | [-30;30] | ↑ PD (UV) |
|  | Khan U. | 2021, paper | Retrospective analysis of medical records (RWE) | USA | 01/2011 - 12/2018 | 414 | Advanced or Metastatic | Mainly Anti-PD-1 | Mainly monotherapy | Multiple | 129 (31%) 143 (35%) | [-84;0] [0;84] | ↓ ORR (MV), ↑ PD (UV) on [0;42] |
|  | Masini | 2019, abstract | Retrospective analysis of medical records (RWE) | Italy | / | 169 | Advanced | Mainly Anti-PD-1 | Monotherapy | / | 59 (35%) | [0;∞[ | ↑ OS (UV) |
|  | Ng Wei Qi | 2021, poster | Retrospective analysis of medical records (RWE) | Ireland | 01/2014 - 12/2020 | 44 | / | Anti-PD-1 | Monotherapy | / | 9 (20%) | [-60;30] | ↓ OS & ↑ PFS (UV), ↓ PD (UV) |
|  | Pérez-Ruiz | 2020, paper | Retrospective analysis of medical records (RWE) | Spain | 2015 - Unknown | 253 | Multiple | Mainly Anti-PD-1 | / | Multiple | 53 (20.5%) | [-60;30] | ↓ OS (UV) |
|  | *Pinato* | 2019, paper | Retrospective analysis of medical records entered into a prospectively maintained database (RWE) | UK | 01/2015 - 01/2018 | 39 | Mainly metastatic | / | / | Multiple | 6 (15.4%) | [-30;0] | ↓ OS (UV) |
|  | *Pinato* | 2019, paper | Retrospective analysis of medical records entered into a prospectively maintained database (RWE) | UK | 01/2015 - 01/2018 | 196 | Mainly metastatic | / | / | Multiple | 26 (13.3%) 66 (33.7%) | [-30;0] [0;∞[ | ↓ ORR (UV), ↑PD (UV) ↑ PD (UV) |
|  | Sen | 2018, letter to the editor | Retrospective analysis of medical records from patients who were prospectively enrolled in clinical trials | USA | 01/2013 - 11/2015 | 172 | Advanced | ICI | Multiple | / | 19 (11%) 14 (8.1%) | [-30;0] [-60;-31] | ↓ OS & ↑ PFS (UV), ↓ PD (UV) ↓ OS & ↑ PFS (UV), ↓ PD (UV) |
|  | *Spakowicz* | 2020, paper | Retrospective analysis of medical records (RWE) | USA | 2011 - 2017 | 225 | Mainly Stage IV | ICI | / | / | 89 (39.6%) | [-28;28] | ↓ OS (UV) |
|  | Tinsley | 2019, paper | Retrospective analysis of medical records (RWE) | UK | 01/2015 - 04/2017 | 291 | Advanced | / | / | Multiple | 92 (32%) | [-14;42] | ↓ OS & ↑ PFS (MV) |
|  | Vick | 2020, poster | Retrospective analysis of medical records (RWE) | USA | / | 216 | Mainly Stage IV | Mainly Anti-PD-1 | / | / | 81 (37.5%) | [-180;0] | ↓ OS & ↑ PFS (UV) |
|  | Vitorino | 2021, poster | Retrospective analysis of medical records (RWE) | Portugal | 01/2016 - 06/2020 | 114 | / | Mainly Anti-PD-1 | / | / | 24 (21%) | [-30;0] | ↑ OS & ↑ PFS (UV), ↓ ORR (UV) |

Of note, the data reported above (number of patients, ABX exposure TW) correspond to survival outcomes unless specified otherwise.


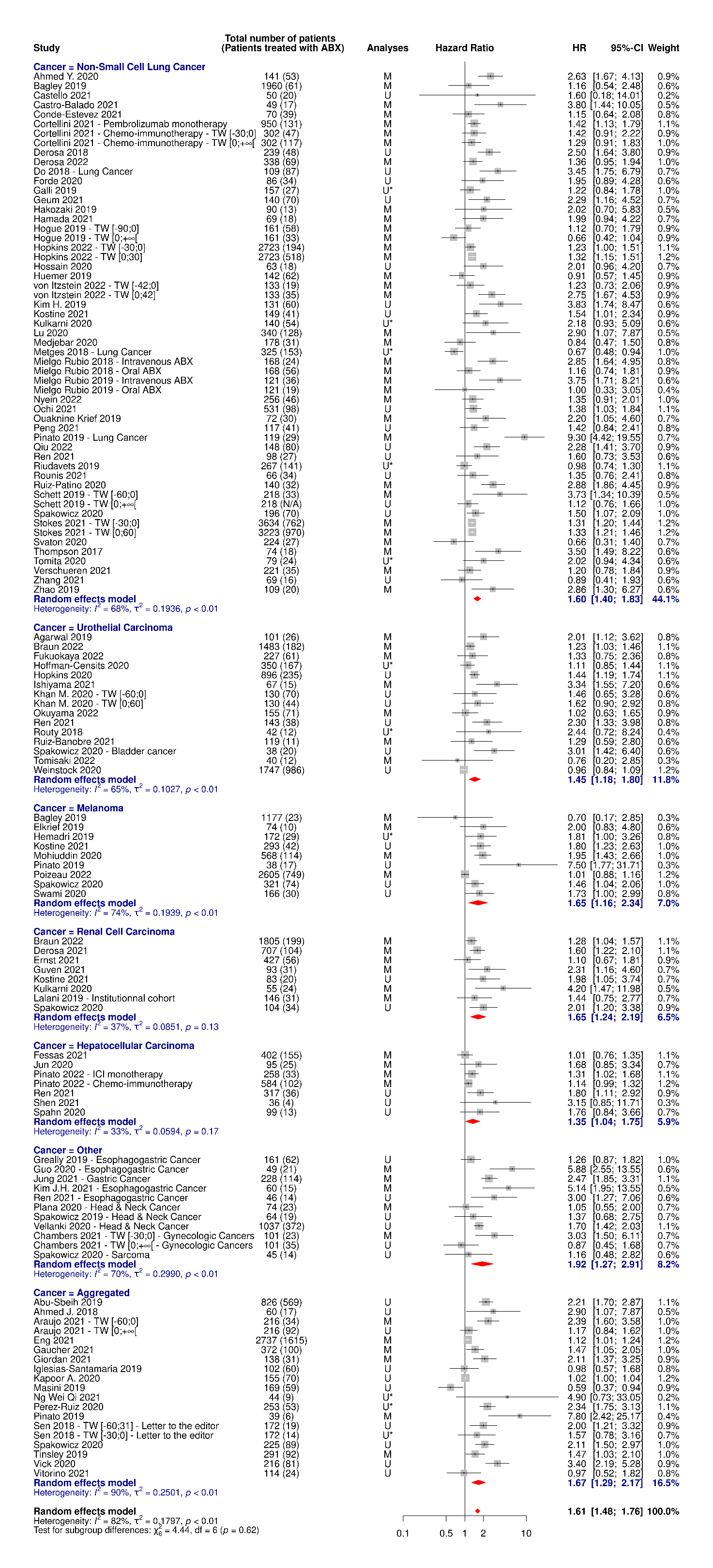


1

Worse OS

Better OS

**Supplementary Figure 2: Forest plot of hazard ratios for overall survival of patients diagnosed with cancer and exposed to antibiotics versus not exposed to antibiotics around immune checkpoint inhibitor treatment initiation, according to the cancer type.** ABX, Antibiotic; CI, Confidence Interval; HR, Hazard Ratio; M, Multivariate; N/A, Not Available; TW, Time Window; U, Univariate; U*, Univariate, HR estimated from Kaplan-Meier curve.

First part of the figure


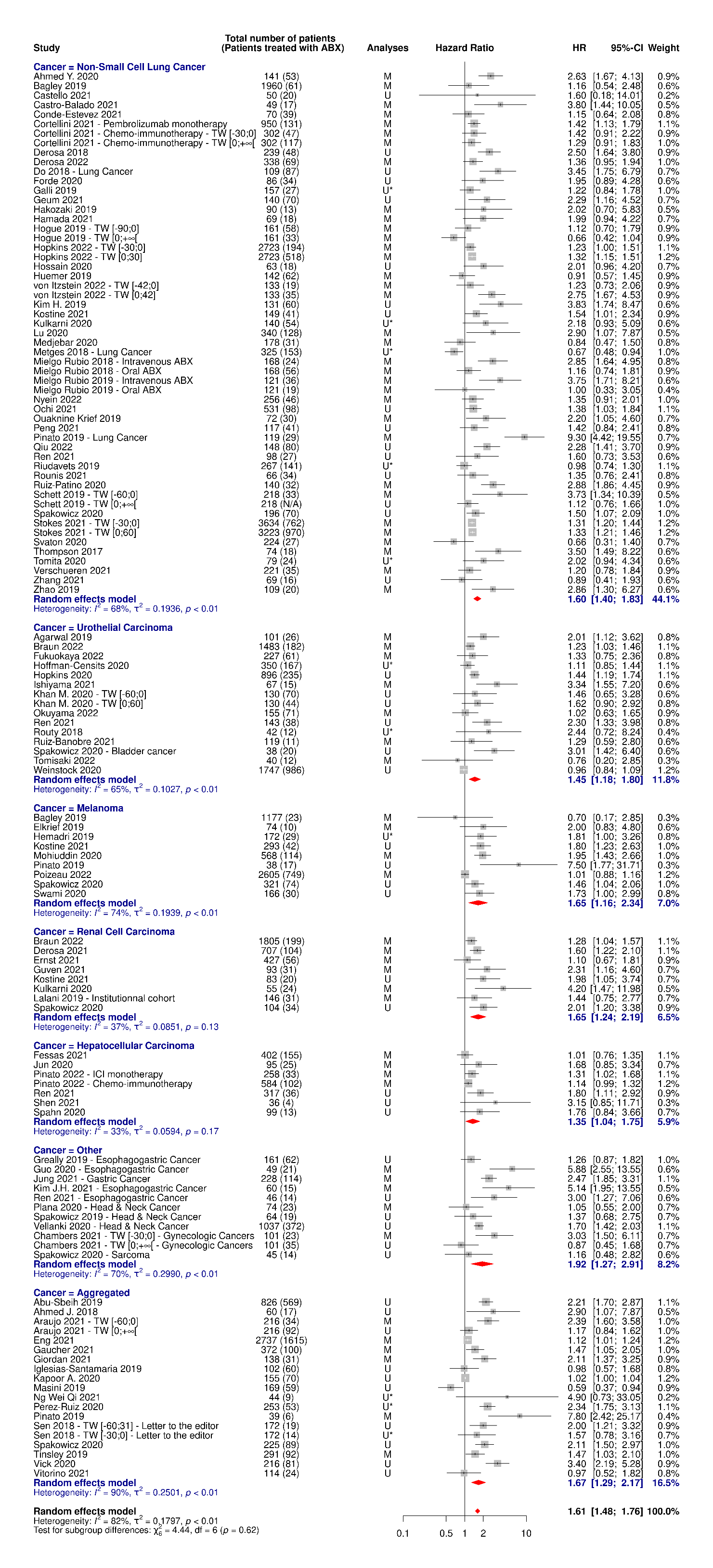


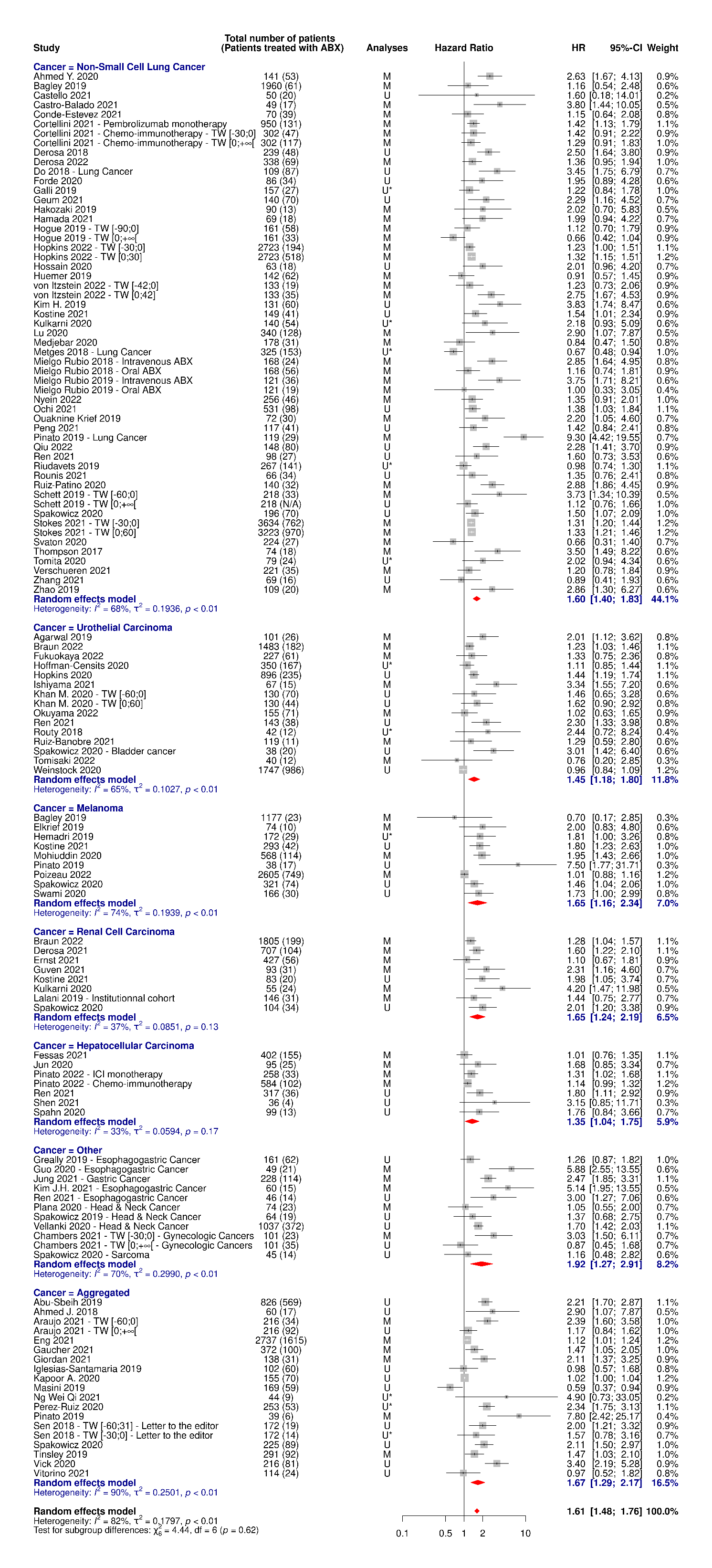


1

Worse OS

Better OS

Second part of the figure


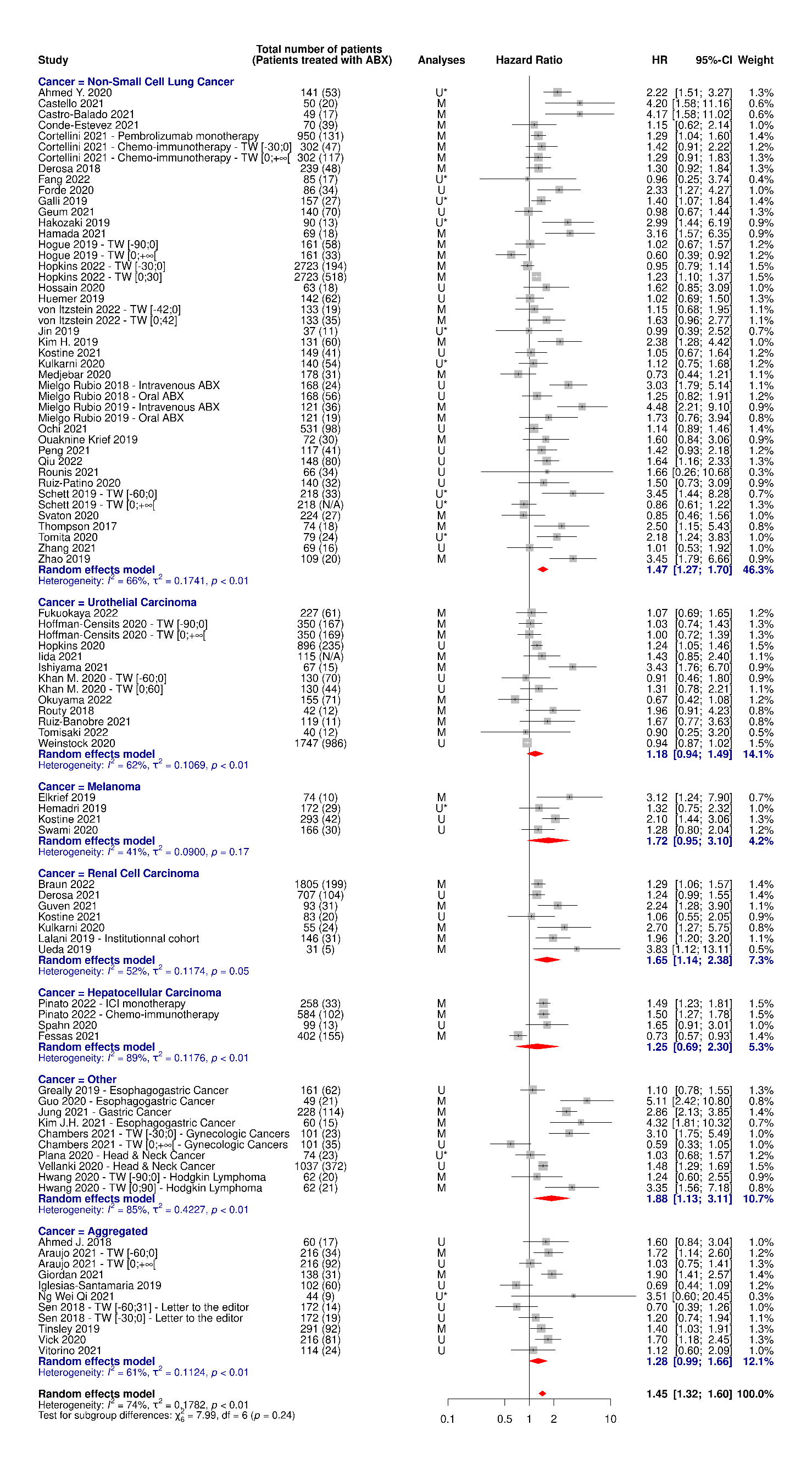


1

Worse PFS

Better PFS

**Supplementary Figure 3: Forest plot of hazard ratios for progression-free survival of patients diagnosed with cancer and exposed to antibiotics versus not exposed to antibiotics around immune checkpoint inhibitor treatment initiation, according to the cancer type.** ABX, Antibiotic; CI, Confidence Interval; HR, Hazard Ratio; M, Multivariate; N/A, Not Available; TW, Time Window; U, Univariate; U*, Univariate, HR estimated from Kaplan-Meier curve.

First part of the figure


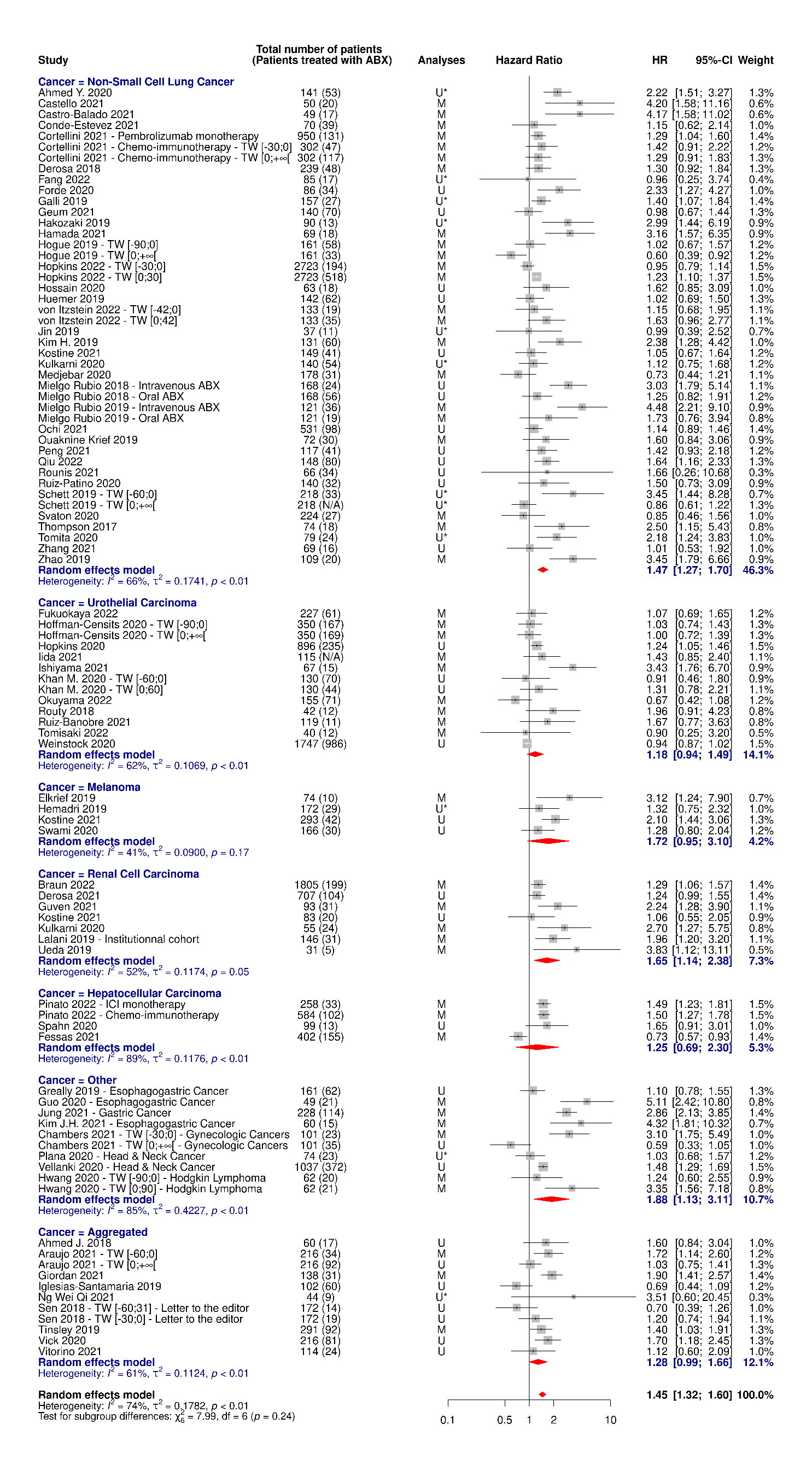

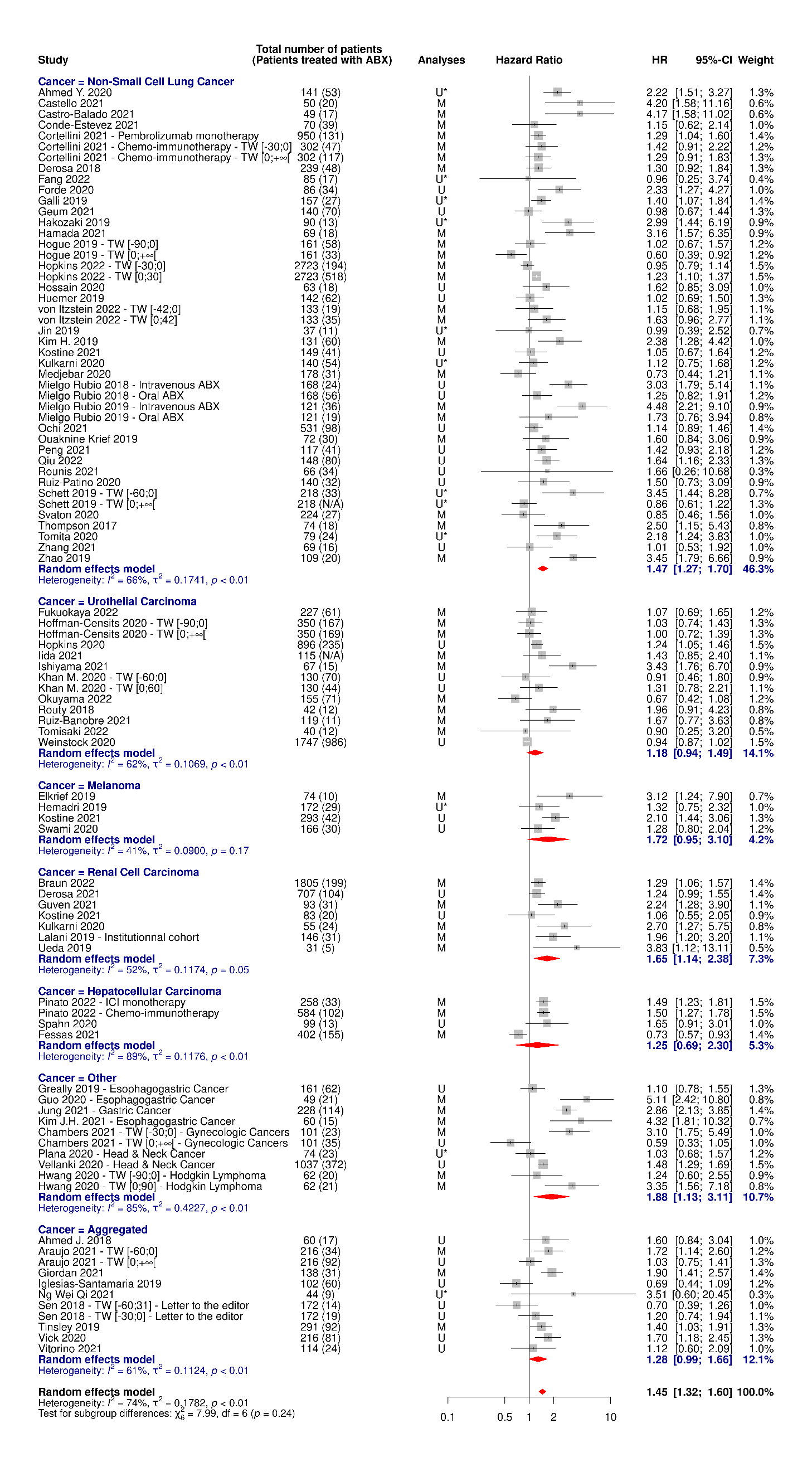


1

Worse PFS

Better PFS

Second part of the figure


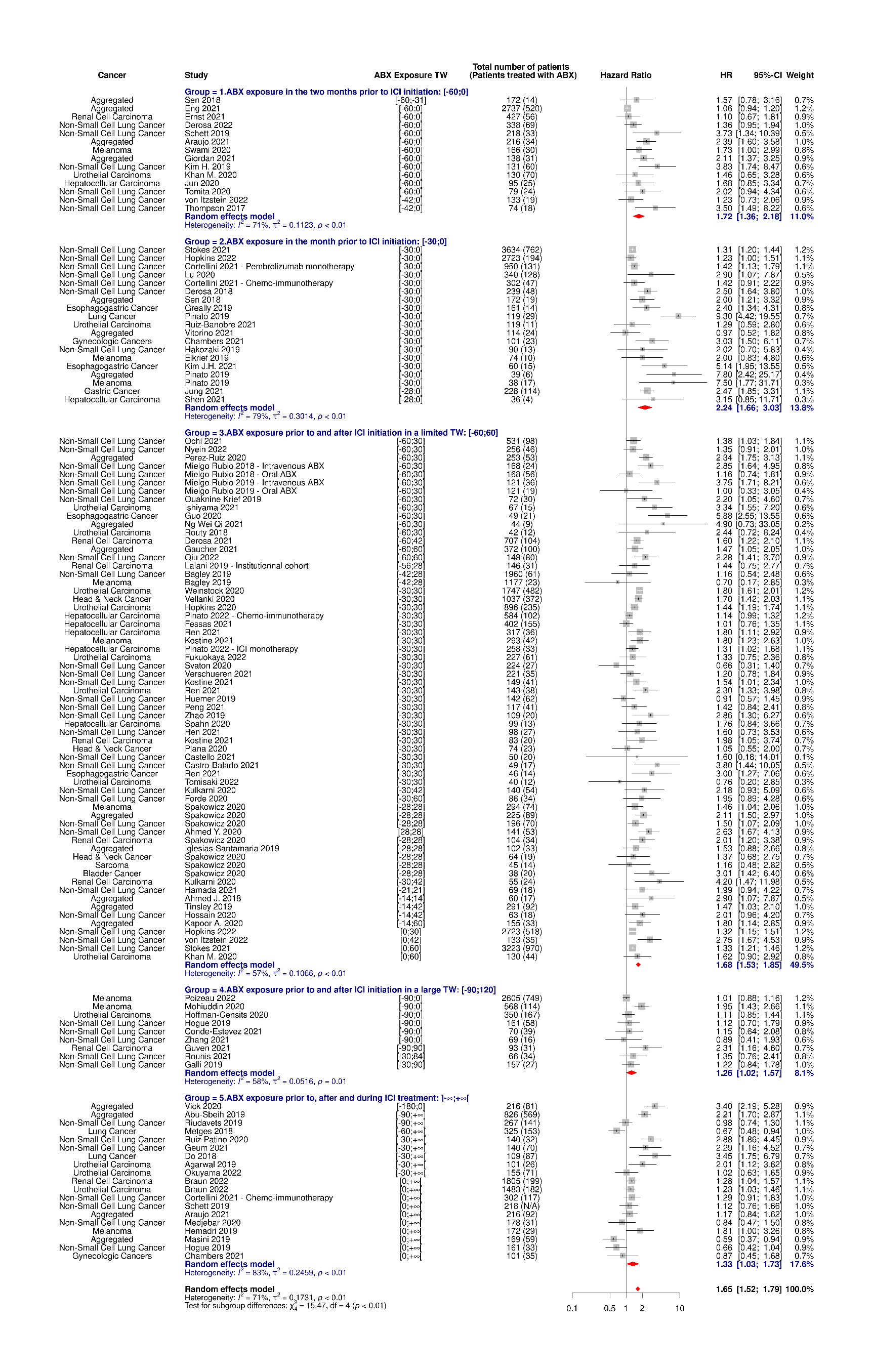


1

Worse OS

Better OS

**Supplementary Figure 4: Forest plot of hazard ratios for overall survival of patients diagnosed with cancer and exposed to antibiotics versus not exposed to antibiotics around immune checkpoint inhibitor treatment initiation, according to the antibiotic exposure time window.** ABX, Antibiotic; CI, Confidence Interval; HR, Hazard Ratio; M, Multivariate; N/A, Not Available; TW, Time Window; U, Univariate; U*, Univariate, HR estimated from Kaplan-Meier curve.

First part of the figure

**
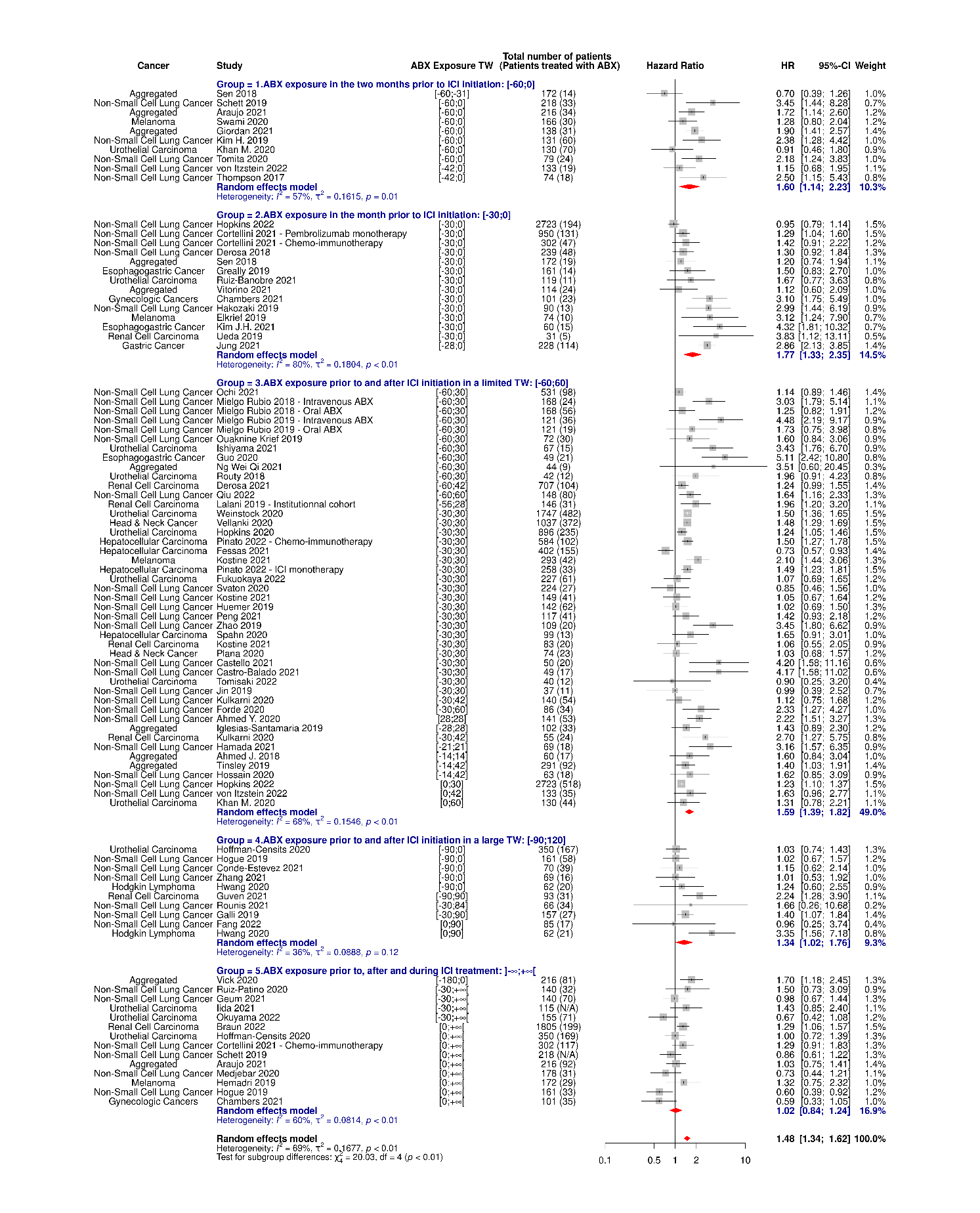
**
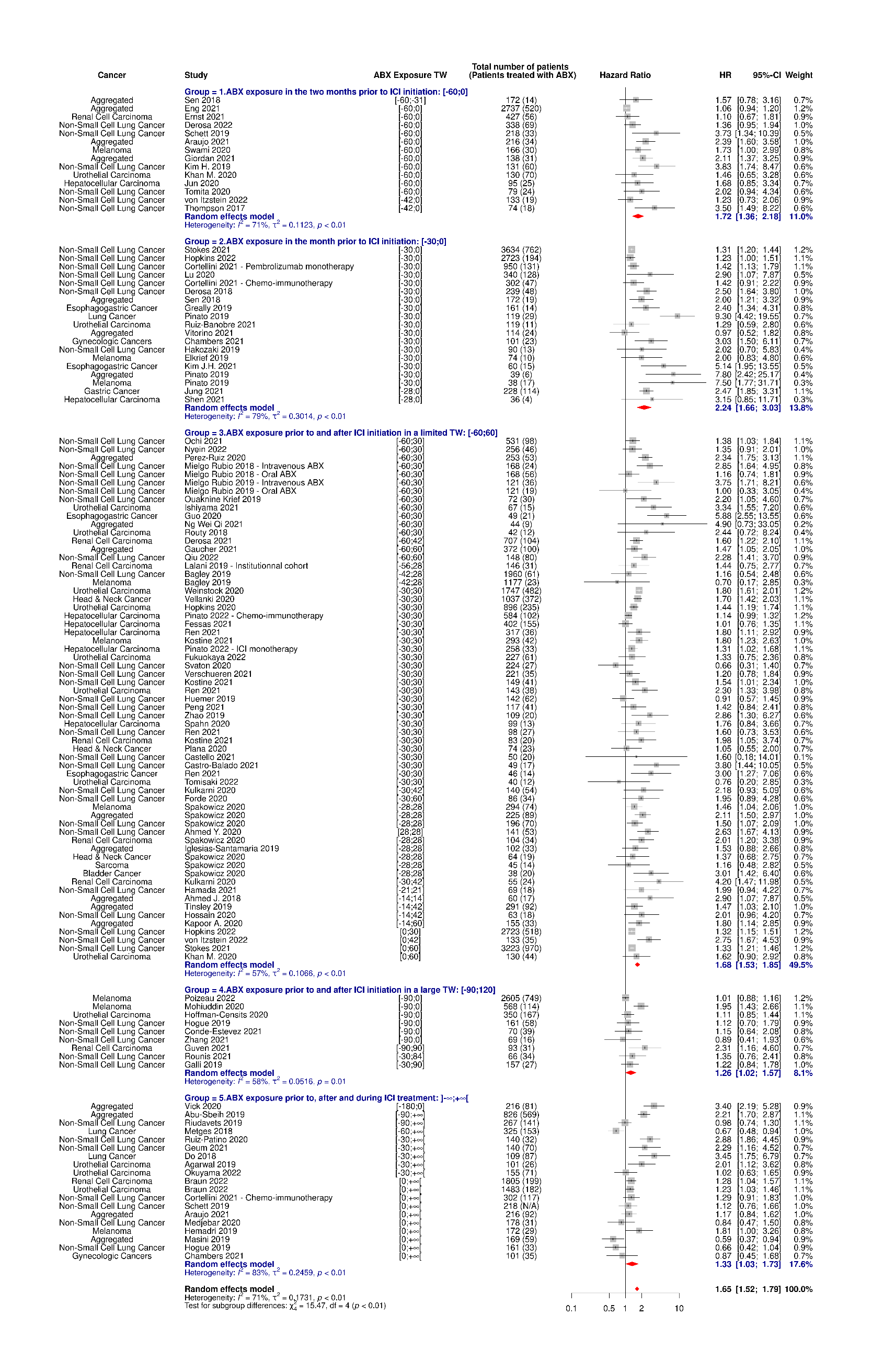


1

Worse OS

Better OS

Second part of the figure

**
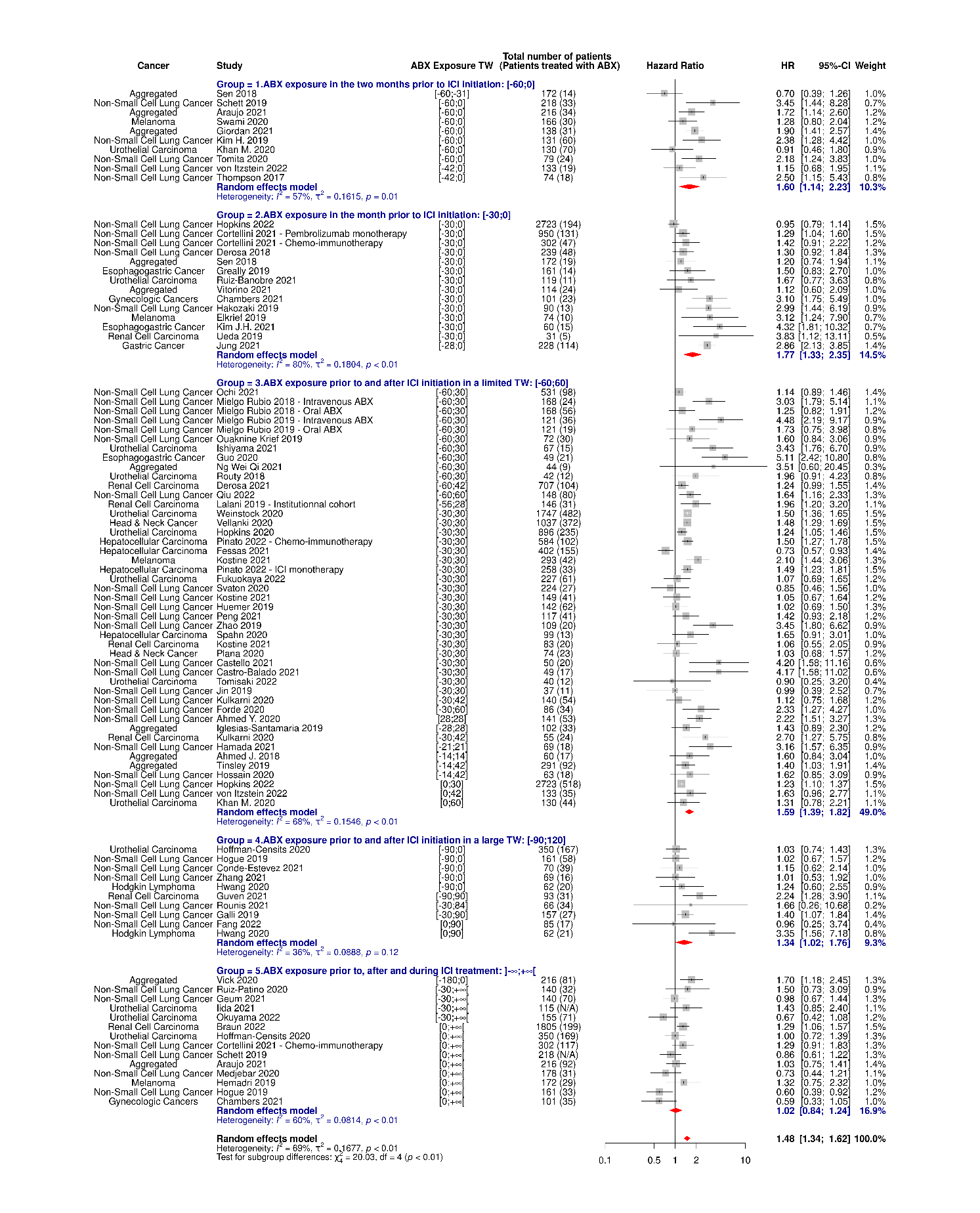
**

1

Worse PFS

Better PFS

**Supplementary Figure 5: Forest plot of hazard ratios for progression-free survival of patients diagnosed with cancer and exposed to antibiotics versus not exposed to antibiotics around immune checkpoint inhibitor treatment initiation, according to the antibiotic exposure time window.** ABX, Antibiotic; CI, Confidence Interval; HR, Hazard Ratio; M, Multivariate; N/A, Not Available; TW, Time Window; U, Univariate; U*, Univariate, HR estimated from Kaplan-Meier curve.

First part of the figure

**
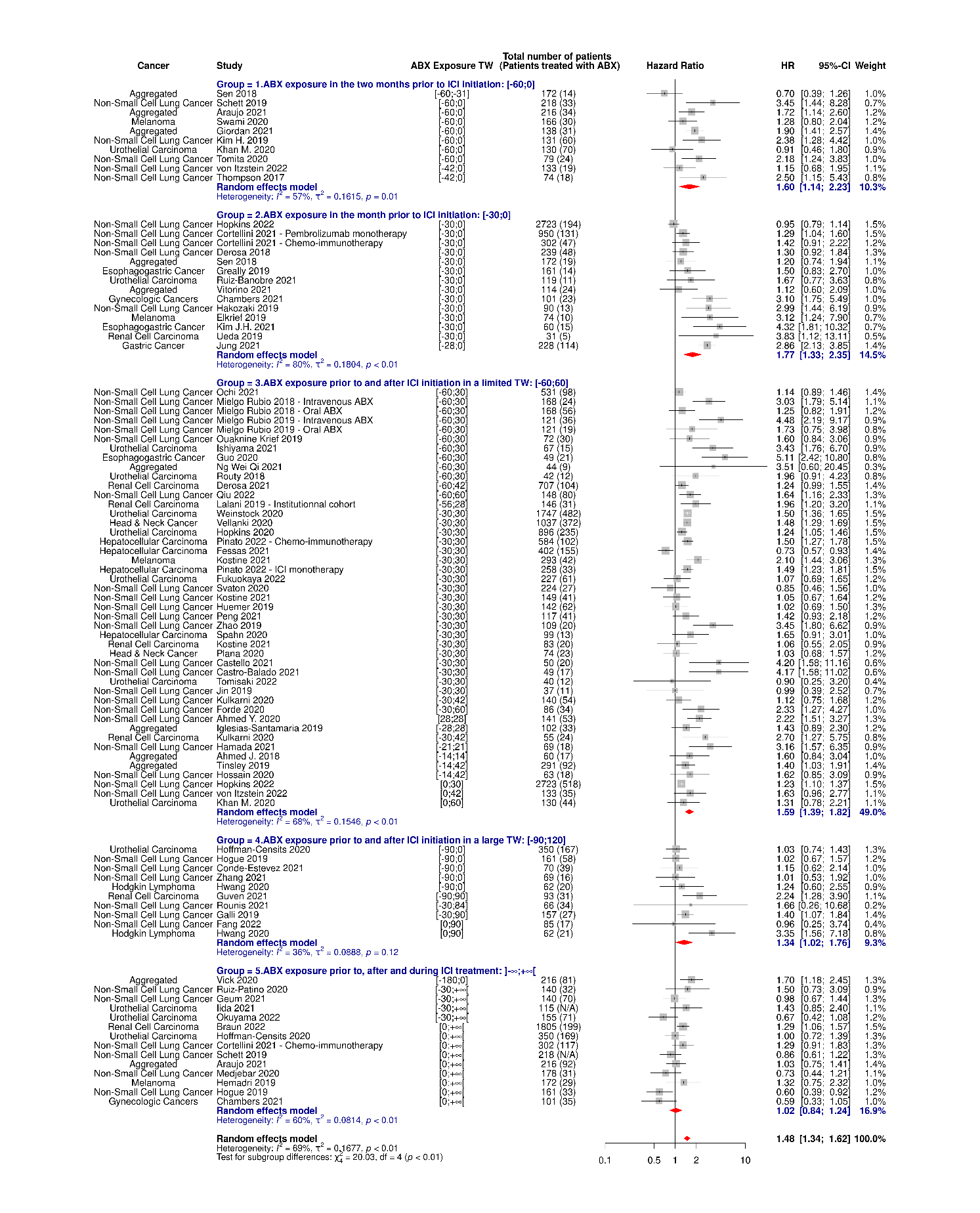

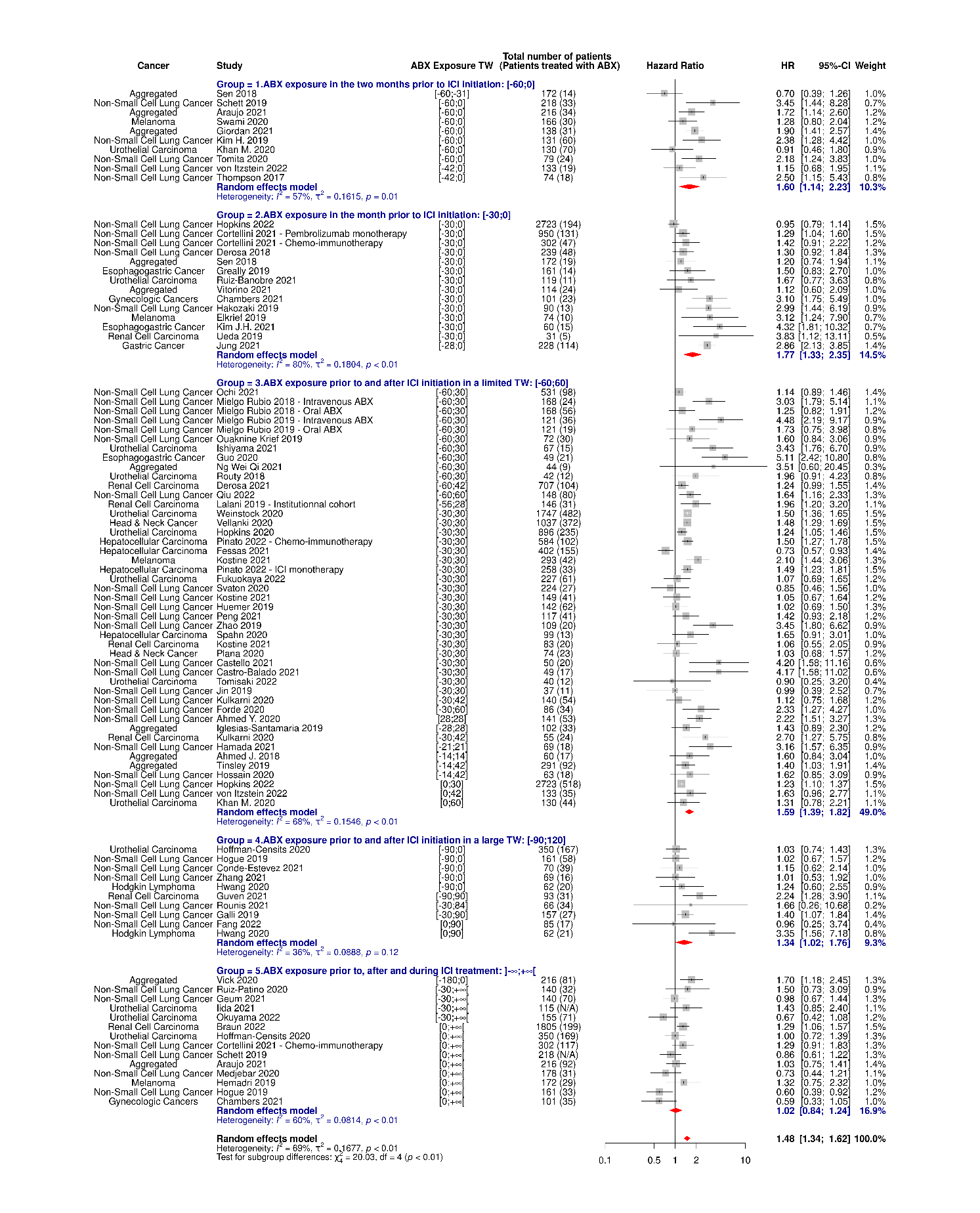
**

1

Worse PFS

Better PFS

Second part of the figure

**
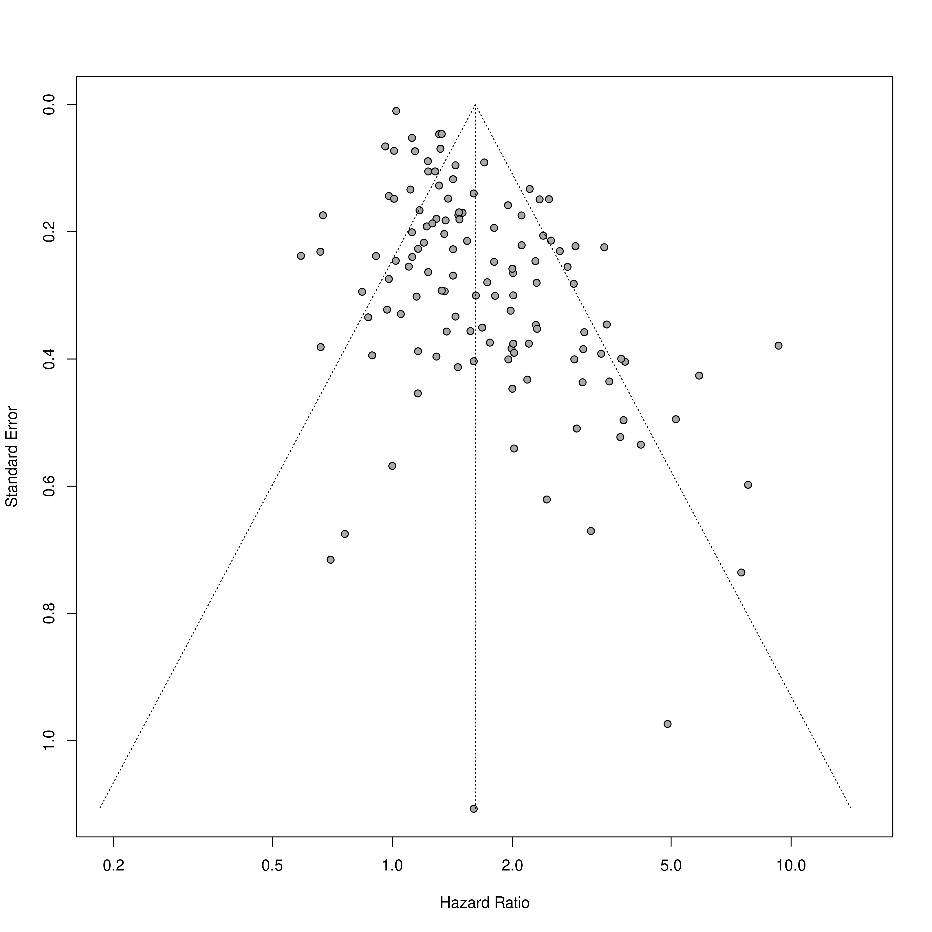
**

**Supplementary Figure 6: Funnel plot for hazard ratios for overall survival of patients diagnosed with cancer and exposed to antibiotics versus not exposed to antibiotics around immune checkpoint inhibitor treatment initiation.**

**
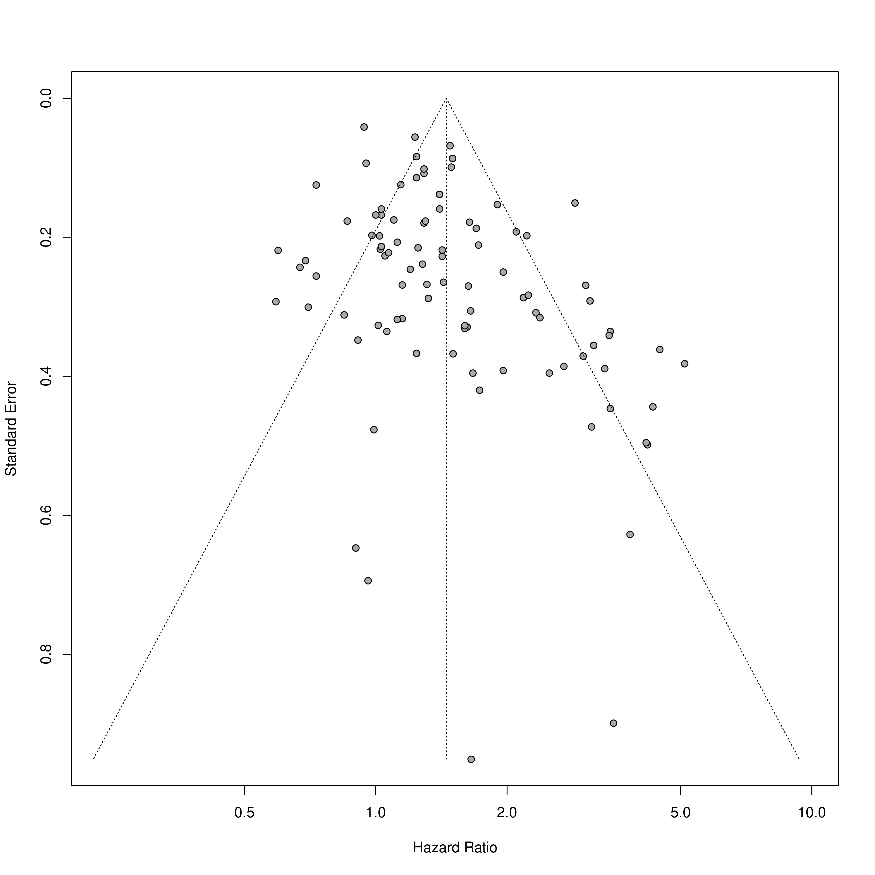
**

**Supplementary Figure 7: Funnel plot for hazard ratios for progression-free survival of patients diagnosed with cancer and exposed to antibiotics versus not exposed to antibiotics around immune checkpoint inhibitor treatment initiation.**

**
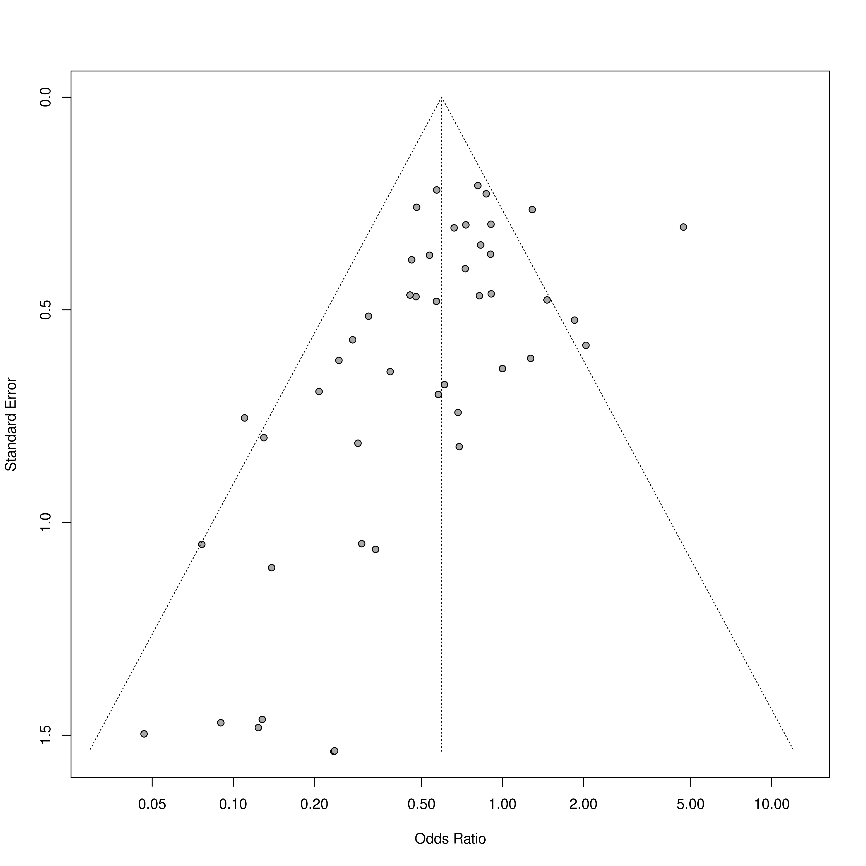
**

**Supplementary Figure 8: Funnel plot for odds ratios for objective response rate of patients diagnosed with cancer and exposed to antibiotics versus not exposed to antibiotics around immune checkpoint inhibitor treatment initiation.**

**
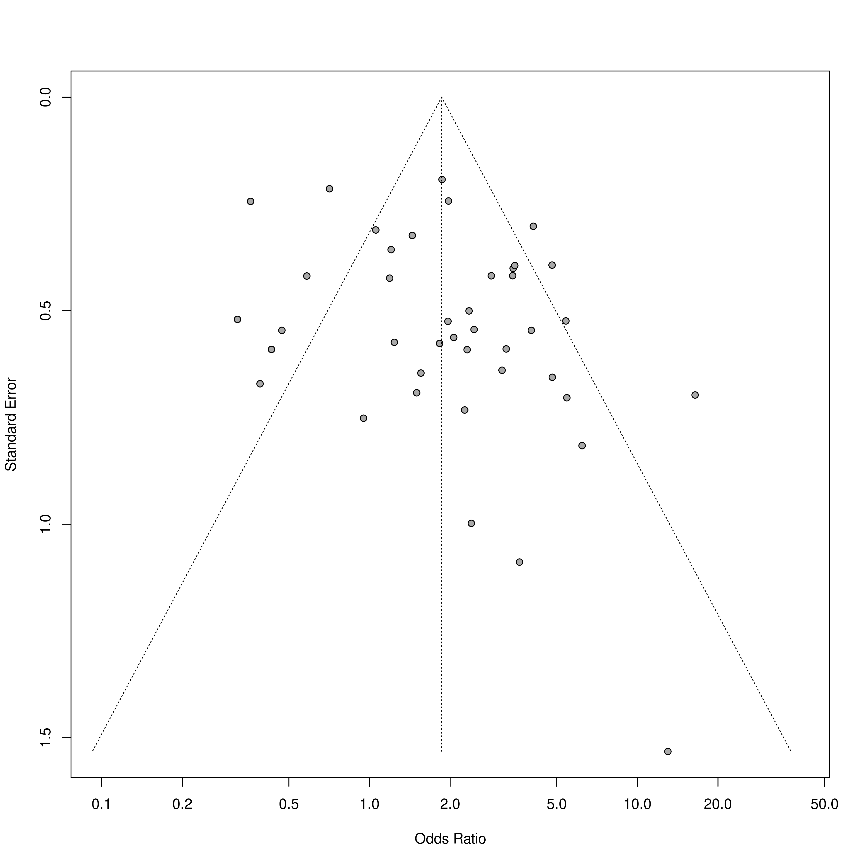
**

**Supplementary Figure 9: Funnel plot for odds ratios for progressive disease rate of patients diagnosed with cancer and exposed to antibiotics versus not exposed to antibiotics around immune checkpoint inhibitor treatment initiation.**


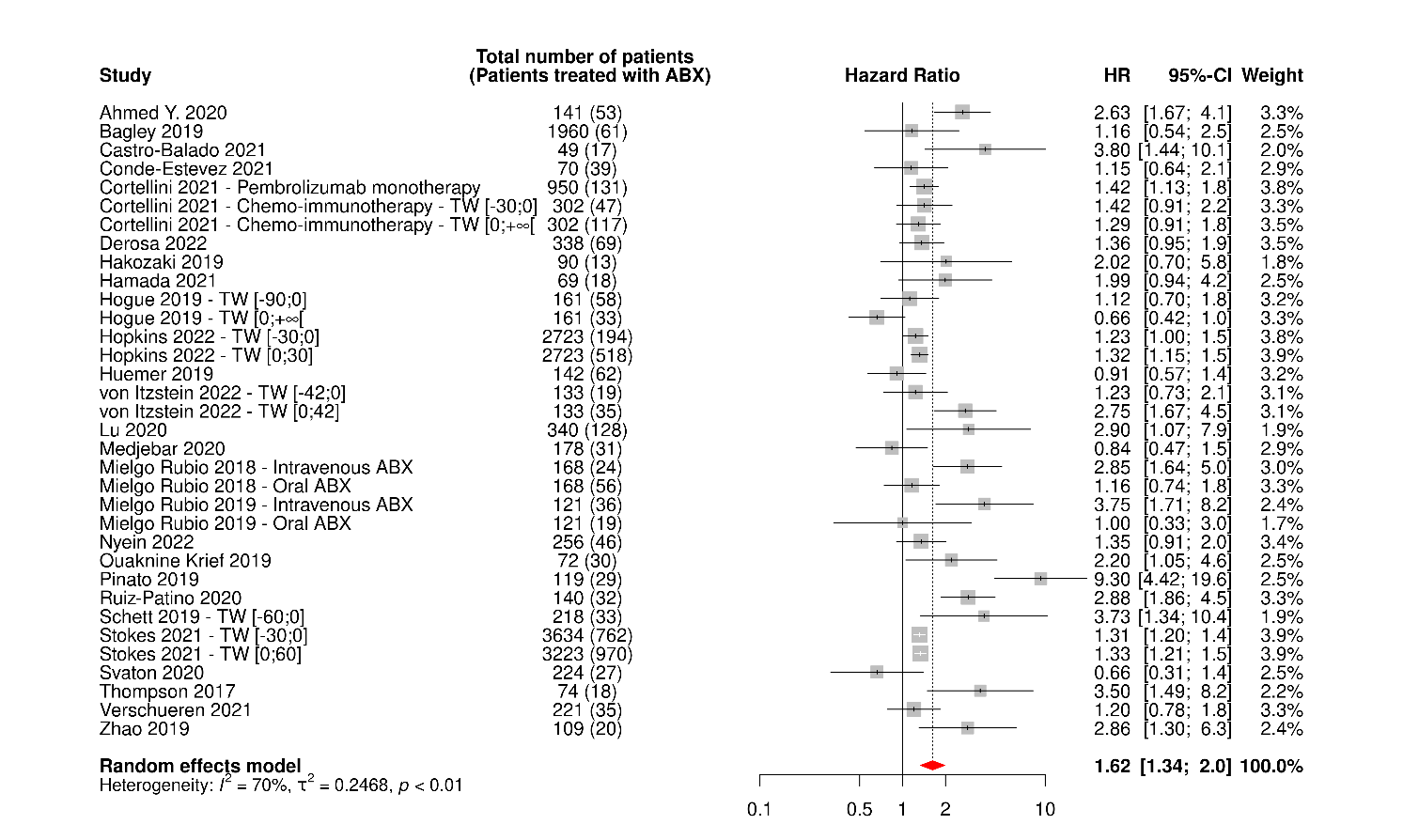


Worse OS

Better OS

**Supplementary Figure 10: Forest plot of hazard ratios yielded from multivariate analyses for - overall survival of patients diagnosed with non-small cell lung cancer exposed to antibiotics versus not exposed to antibiotics around immune checkpoint inhibitor treatment initiation.** ABX, Antibiotic; CI, Confidence Interval; HR, Hazard Ratio; TW, Time Window.
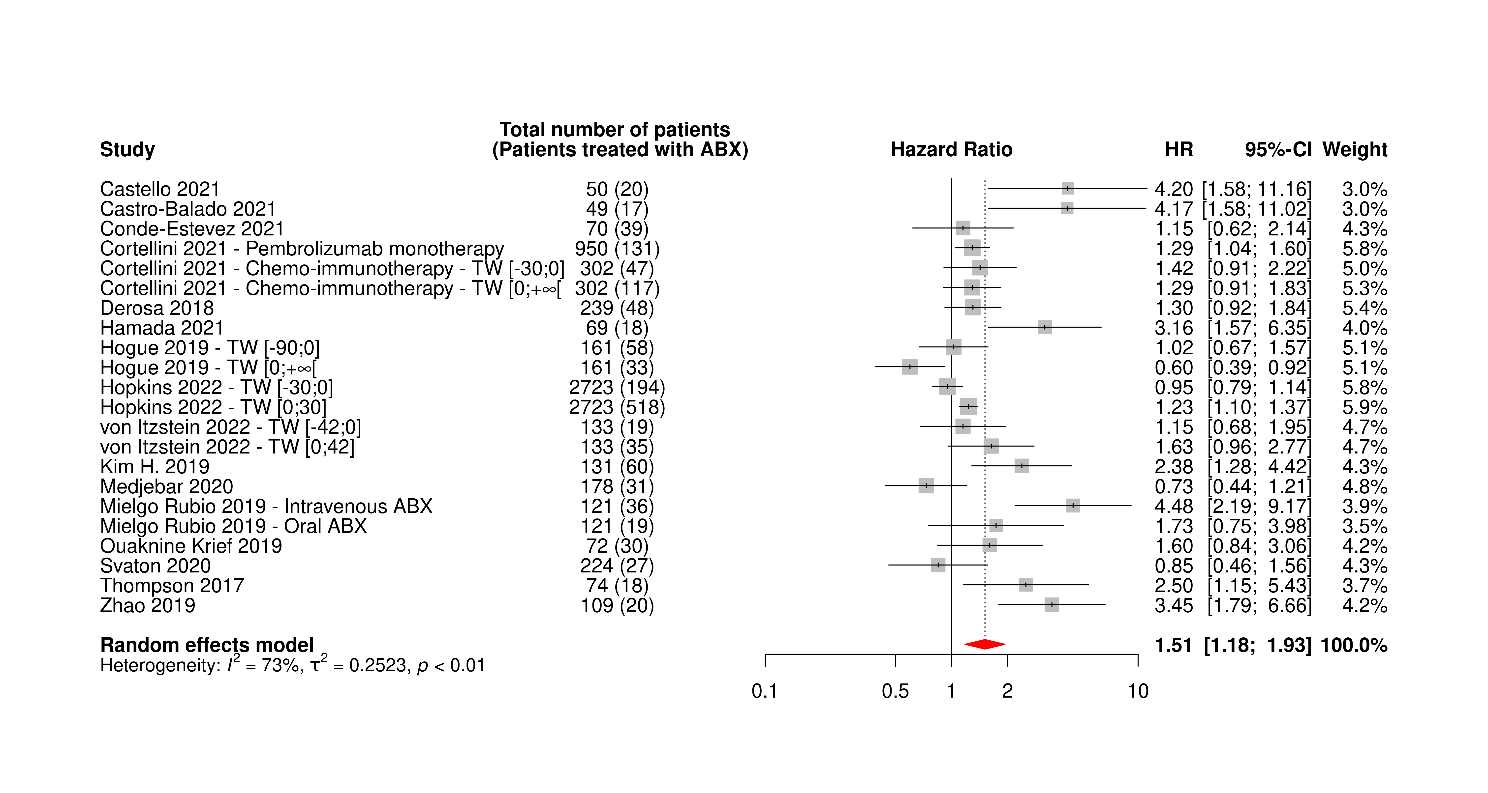


Worse PFS

Better PFS

**Supplementary Figure 11: Forest plot of hazard ratios yielded from multivariate analyses for progression-free survival of patients diagnosed with non-small cell lung cancer exposed to antibiotics versus not exposed to antibiotics around immune checkpoint inhibitor treatment initiation.** ABX, Antibiotic; CI, Confidence Interval; HR, Hazard Ratio; TW, Time Window.

**
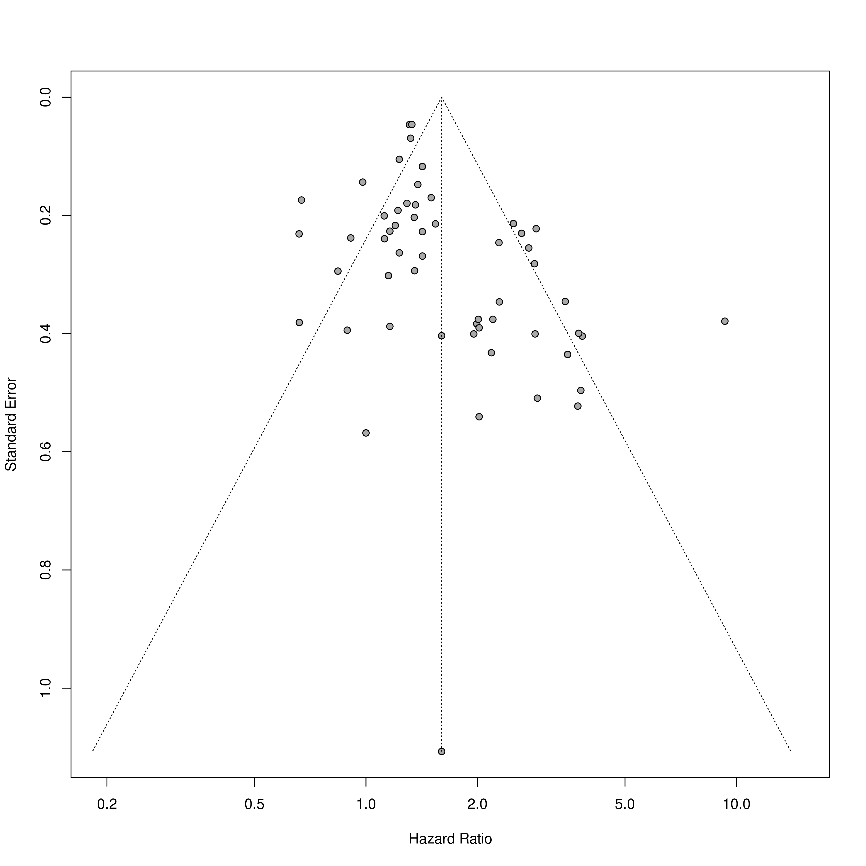
**

**Supplementary Figure 12: Funnel plot for hazard ratios for overall survival of patients diagnosed with non-small cell lung cancer and exposed to antibiotics versus not exposed to antibiotics around immune checkpoint inhibitor treatment**

**
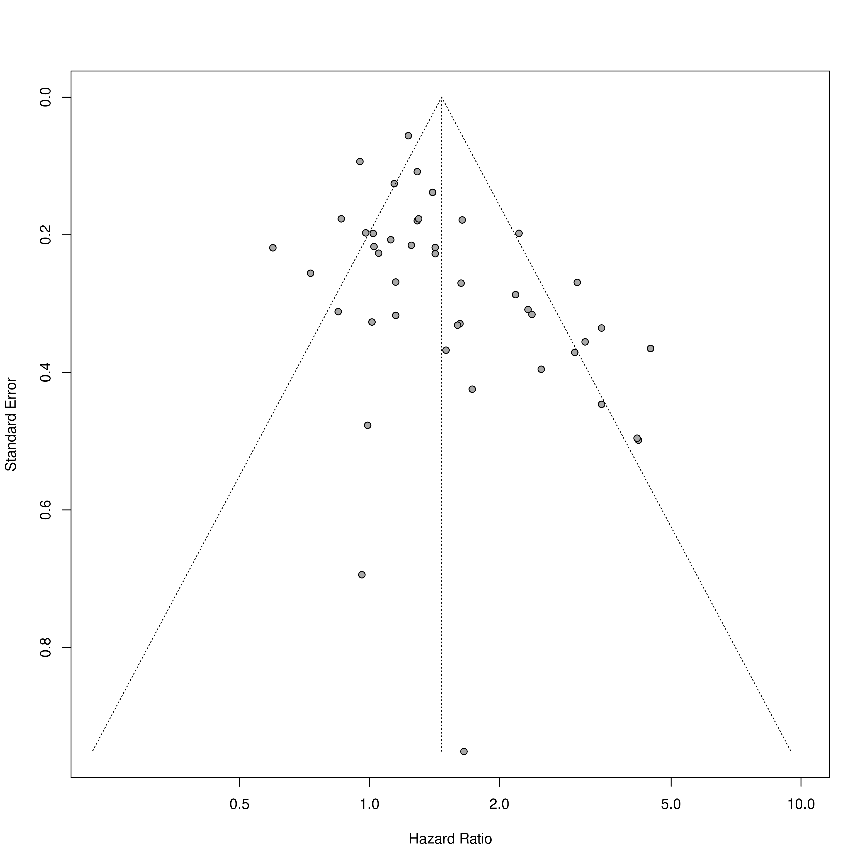
**

**Supplementary Figure 13: Funnel plot for hazard ratios for progression-free survival of patients diagnosed with non-small cell lung cancer and exposed to antibiotics versus not exposed to antibiotics around immune checkpoint inhibitor treatment**

**
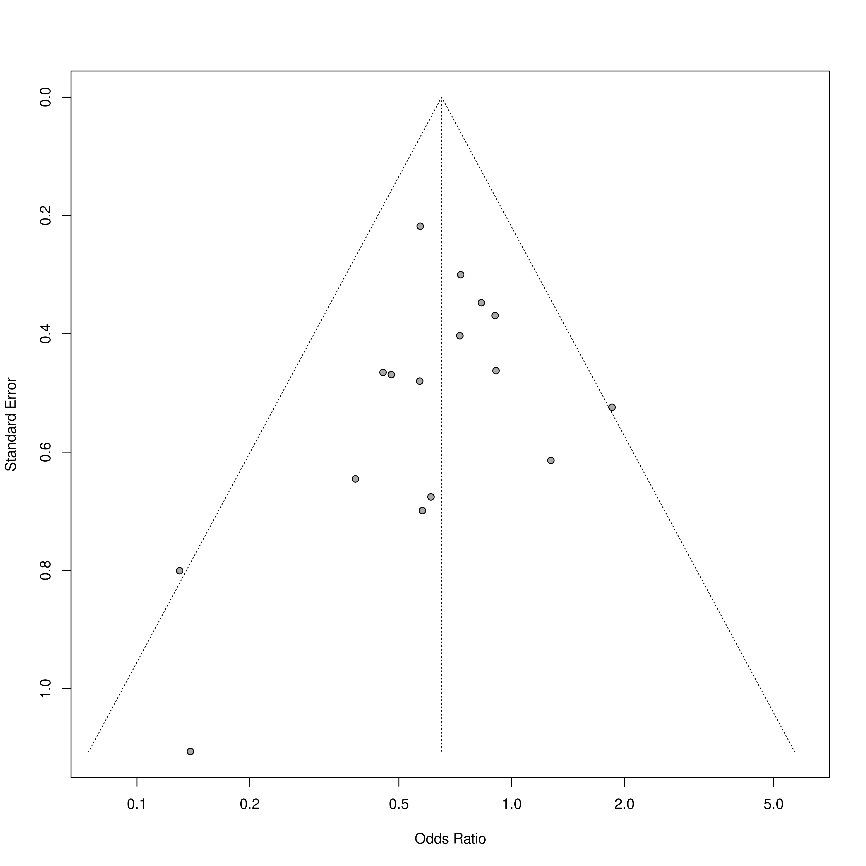
**

**Supplementary Figure 14: Funnel plot for odds ratios for objective response rate of patients diagnosed with non-small cell lung cancer and exposed to antibiotics versus not exposed to antibiotics around immune checkpoint inhibitor treatment**

**
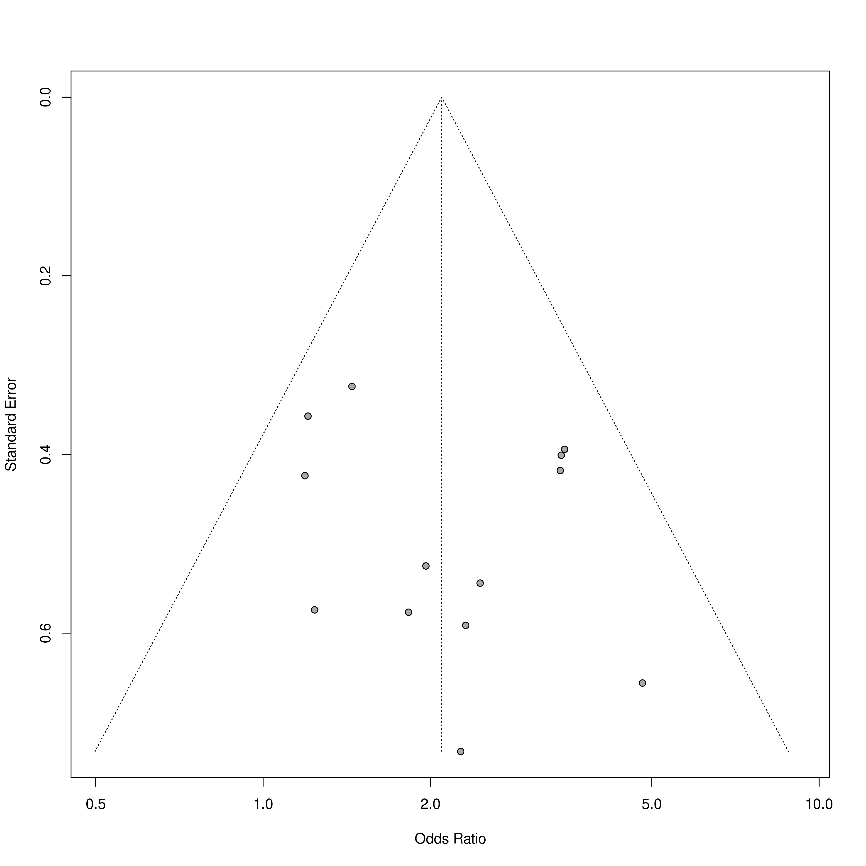
**

**Supplementary Figure 15: Funnel plot for odds ratios for progressive disease rate of patients diagnosed with non-small cell lung cancer and exposed to antibiotics versus not exposed to antibiotics around immune checkpoint inhibitor treatment**
